# Synergistic effects of neuroprotective drugs with intravenous recombinant tissue plasminogen activator in acute ischemic stroke: A Bayesian network meta-analysis

**DOI:** 10.1371/journal.pone.0311231

**Published:** 2024-12-02

**Authors:** Chun Dang, Qinxuan Wang, Yijia Zhuang, Qian Li, Yaoheng Lu, Ying Xiong, Li Feng

**Affiliations:** 1 Department of Periodical Press/Chinese Evidence-Based Medicine Center, West China Hospital, Sichuan University, Chengdu, China; 2 West China Hospital, West China School of Medicine, Sichuan University, Chengdu, China; 3 Department of Neurology, The Second Affiliated Hospital of Harbin Medical University, Harbin, China; 4 Department of General Surgery, Chengdu Integrated Traditional Chinese Medicine and Western Medicine Hospital, Chengdu, China; 5 Department of General Surgery and Regenerative Medicine Research Center, West China Hospital, Sichuan University, Chengdu, China; College of Medical Sciences, NEPAL

## Abstract

Neuroprotective drugs as adjunctive therapy for adults with acute ischemic stroke (AIS) remains contentious. This study summarizes the latest evidence regarding the benefits of neuroprotective agents combined with intravenous recombinant tissue plasminogen activator (rt-PA) intravenous thrombolysis. This study conducted a structured search of PubMed, the Cochrane Library, EMBASE, Wanfang Data, and CNKI databases from their inception to March 2024. Grey literature was also searched. The outcomes included efficacy (National Institutes of Health Stroke Scale (NIHSS) score and Barthel Index (BI) score) and safety (rate of adverse reactions). A total of 70 randomized controlled trials were selected for this network meta-analysis (NMA), encompassing 4,140 patients with AIS treated using different neuroprotective agents plus RT-PA, while 4,012 patients with AIS were in control groups. The top three treatments for NIHSS scores at the 2-week follow-up were Edaravone Dexborneo with 0.9 mg/kg rt-PA, Edaravone with 0.9 mg/kg rt-PA, and HUK with 0.9 mg/kg rt-PA. HUK with 0.9 mg/kg rt-PA, Dl-3n-butylphthalide with 0.9 mg/kg rt-PA, and Edaravone Dexborneo with 0.9 mg/kg rt-PA were ranked the top three for BI scores at the 2-week follow-up. The top three treatments with the lowest adverse effect rates were 0.6 mg/kg rt-PA, HUK with 0.9 mg/kg rt-PA, and Edaravone Dexborneo with 0.9 mg/kg rt-PA due to their excellent safety profiles. Compared to rt-PA alone, the combination treatments of Edaravone+rt-PA, Edaravone Dexborneol+rt-PA, HUK+rt-PA, Dl-3n-butylphthalide+rt-PA, and Ganglioside GM1+rt-PA have shown superior efficacy. This NMA suggest that combination therapies of neuroprotective agents and rt-PA can offer better outcomes for patients with AIS. The results support the potential integration of these combination therapies into standard AIS treatment, aiming for improved patient outcomes and personalized therapeutic approaches.

## Introduction

Stroke is a severe condition caused by acute localized vascular injury in the brain, the second leading cause of death globally [1]. Stroke brings a substantial burden on patients, and healthcare systems worldwide.

Recombinant tissue plasminogen activator (rt-PA) intravenous thrombolysis (IVT) is currently the only thrombolytic therapy approved by the Food and Drug Administration for treating acute ischemic stroke (AIS) within 4.5 hours of symptom onset [2]. This treatment works by breaking down fibrin-rich clots that obstruct blood flow, thereby facilitating reperfusion [3].

Neuroprotection remains a crucial goal in AIS therapy. Neuroprotective agents are widely used in Asian countries and are recommended for the treatment of AIS by both Chinese and Japanese stroke care guidelines. Notable neuroprotective agents include Edaravone, Edaravone Dexborneol, HUK, and Dl-3n-butylphthalide [4, 5]. Therapeutic approaches have focused on reducing excitotoxicity, inhibiting calcium influx across cell membranes, and mitigating damage caused by inflammation, free radicals, and intracellular enzymes [6]. Although the internationally recommended dosage for rt-PA is 0.9 mg/kg, the Japanese stroke care guidelines recommend a lower dose of 0.6 mg/kg for the treatment of AIS. Accordingly, the dose of IV-rt-PA in Asia remains controversial [7].

However, the efficacy of neuroprotectants as adjunct therapy compared to monotherapy with rt-PA remains contentious [8]. Studies indicate that certain neuroprotectants, when used in conjunction with rt-PA, can enhance neurological recovery in patients, although the efficacy varies across different combination therapies. Some research demonstrates that specific combinations can significantly reduce the area of brain damage and decrease long-term disability rates. Conversely, other studies report no significant difference in efficacy between neuroprotectants and placebo. Therefore, it is necessary to conduct indirect comparisons of various neuroprotectants as adjunct therapy with rt-PA, assessing both clinical efficacy and safety [8–13].

Network meta-analysis (NMA) is an advanced technique used to compare multiple intervention options within a systematic review of numerous clinical trials. This approach integrates direct evidence (comparing interventions within the same trials) and indirect evidence (comparing interventions across different trials using a common comparator). Bayesian NMA is based on the principles of Bayesian statistics, differing from traditional meta-analysis. In Bayesian meta-analysis, the results of studies are considered as expressions of uncertainty rather than fixed truths. A significant advantage of Bayesian NMA lies in its ability to simultaneously compare treatments and ascertain their efficacy through posterior probability, aiding in the selection of superior neuroprotective treatments for future stroke therapy. The use of Bayesian NMA in synthesizing evidence is becoming increasingly prevalent, offering valuable insights for making healthcare or policy decisions [14, 15].

In this study, we performed a comprehensive Bayesian NMA to provide a ranking of multiple neuroprotective treatments as adjunct therapy with rt-PA for AIS based on their efficacy and safety.

## Methods

### Search strategy and selection criteria

This NMA is reported in accordance with the Preferred Reporting Items for Systematic Reviews and Meta-Analyses (PRISMA) statement [16]. The study protocol has been registered on PROSPERO (registration number CRD42023439299).

We conducted a structured search of PubMed, the Cochrane Library, EMBASE, Wanfang Data, and the CNKI database from their inception to March 2024. Grey literature was also searched. Keywords used in the search included: 'ischemic stroke', 'acute ischemic stroke', 'AIS', 'cerebral artery occlusion', 'citicoline', 'cerebrolysin', 'Dl-3n-butylphthalide', 'ganglioside GM1', 'edaravone', 'edaravone dexborneol', 'vinpocetine', 'minocycline', 'human urinary kallidinogenase', 'HUK', 'endovascular treatment', 'immunomodulators', 'neuroprotectant therapy', 'neuroprotectant', 'rtPA', 'rt-PA', 'recombinant tissue-type plasminogen activator', 'reperfusion therapy', 'uric acid', 'alteplase', 'immunomodulators', 'intravenous thrombolysis', ' rt-PA', 'neuroprotective agents', 'thrombolysis', and 'prognosis'. We restricted the search to publications in the English and Chinese languages. The specific search strategy is outlined in S1 Table.

### Inclusion and exclusion criteria

Studies included in the NMA met the following criteria: (1) adult patients (≥18 years of age) with AIS who met standard criteria for rt-PA; (2) rt-PA dose was 0.9 mg/kg or 0.6 mg/kg; (3) patients receiving neuroprotective agents plus rt-PA; (4) studies that reported National Institutes of Health Stroke Scale (NIHSS) score, Barthel Index (BI) score, and rate of drug-related adverse reactions; (5) the study was a randomized controlled trial (RCT).

The exclusion criteria were as follows: (1) the study did not meet the diagnostic criteria for AIS; (2) patients receiving endovascular therapy or multiple neuroprotective treatments; (3) patients receiving non-pharmacologic neuroprotection, such as near-infrared laser therapy, hyperbaric oxygen therapy, or hypothermia therapy; (4) non-RCTs.

### Data extraction and quality assessment

During the literature screening process, we initially reviewed the title and abstract of each study. Subsequently, after discarding studies that were clearly irrelevant, we proceeded to a full-text examination to ascertain the suitability of the research for inclusion. The data extraction process involved gathering essential information from the studies. We also collected baseline characteristics of the subjects, such as the number of participants, as well as age, gender. Furthermore, detailed information was collected about the intervention therapy, control therapy, dosage, administration route, and duration of follow-up.

Two reviewers (Chun Dang and Yaoheng Lu) evaluated the risk of bias in each study using the Cochrane Collaboration’s risk of bias assessment tool (RoB 2.0) [17]. Each study was judged to have either ‘low risk of bias,’ ‘high risk of bias,’ or ‘some concerns’ in accordance with the bias evaluation criteria. Any disagreements were settled by a third expert (Qian Li).

### Statistical analysis

Bayesian NMA was performed using random-effect generalized linear models based on the Markov Chain Monte Carlo (MCMC) method [18]. Random effects models were selected in accordance with methodological and clinical heterogeneity. A standard random-effects model was used due to the anticipated variability across different regimens, aiming to yield more conservative estimates of the effects. The *I*^*2*^ statistic was used to evaluate statistical heterogeneity [19]. We utilized mean difference as the statistical effect size for continuous variables, and odds ratio (OR) for binary variables, depending on the type of outcome data. 95% confidence intervals (*CI*) were used as limits. We visualized the collected evidence by creating a network diagram for all outcomes. The ranking of NIHSS scores and BI scores (2-week follow-up) and safety risk associated with different treatments were determined based on the surface under the cumulative ranking (SUCRA) curve [20]. League tables were utilized to encapsulate all potential comparisons within the network, highlighting whether the estimated differences among various regimens exhibited statistical significance. The assessment of model fit was conducted through the calculation of the Deviance Information Criterion (DIC), defined as the sum of the posterior mean of residual deviance and the leverage parameter (pD) [21, 22]. Publication biases were assessed via funnel plots. Pairwise meta-analysis was performed by using Stata, version 17, and NMA within the Bayesian framework was conducted by using R software, version 4.3.1 [23, 24].

## Results

### Study selection

**Fig 1:**
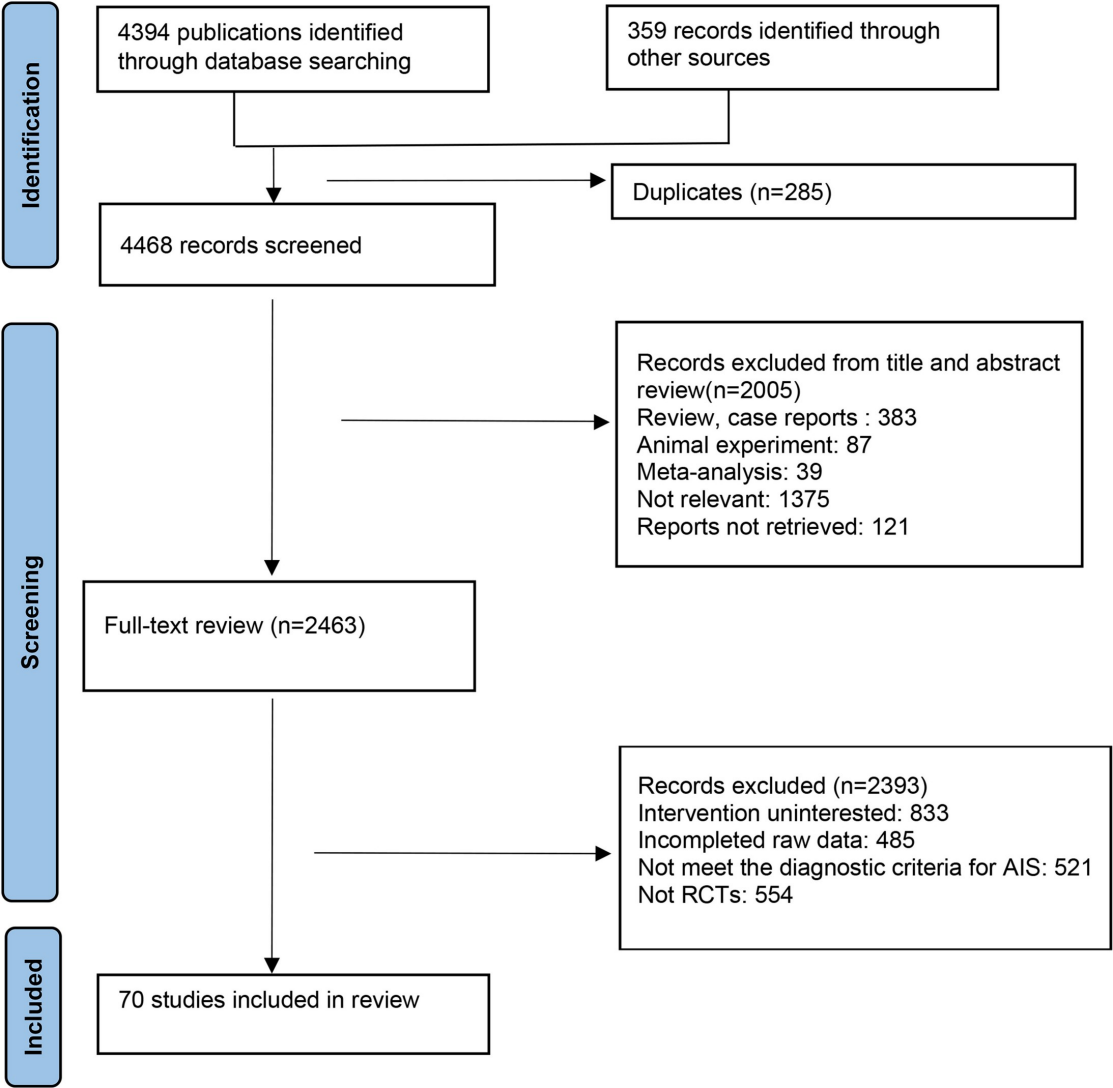
Search results and study selection. This PRISMA-adapted flow diagram presents the retrieved search results from various databases and outlines the literature screening process.

The initial literature search identified 4,394 potentially eligible publications through databases (PubMed, EMBASE, the Cochrane Library, CNKI, and Wanfang Data) and registers, and 359 studies through other methods (Proquest, Open Grey, Google Scholar). Before screening, 285 duplicate records were excluded. Subsequently, publications were eliminated based on reviews, case reports (n = 383), meta-analysis (n = 39), preclinical trials (n = 87), non-relevant publications (n = 1,375), and reports that could not be retrieved (n = 121). Further exclusions were made for studies with incomplete data (n = 491), non-RCTs (n = 554), irrelevant interventions (n = 827), and studies not meeting the diagnostic criteria for AIS (n = 521) in accordance with the inclusion criteria. Ultimately, 70 studies were included, as depicted in Fig 1 [25–94].

### Study and participant characteristics

A total of 70 RCTs were selected for this NMA, encompassing 4,140 patients with AIS who were treated using different neuroprotective agents in combination with rt-PA, while 4,012 patients with AIS were in control groups. The classification of the studies is as follows: 3 RCTs evaluated Cerebrolysin+rt-PA, encompassing 236 patients and 226 controls; 22 RCTs focused on Dl-3n-butylphthalide+rt-PA, involving 1,557 patients and 1,556 controls; 8 RCTs investigated Edaravone Dexborneol+rt-PA with a total of 420 patients and 402 controls; 14 RCTs on Edaravone+rt-PA included 609 patients and 615 controls; 2 RCTs studied Ganglioside GM1+rt-PA with 156 patients and 98 controls; 13 RCTs on HUK+rt-PA comprised 809 patients and 809 controls. 8 RCTs studied 0.9 mg/kg rt-PA with 353 patients and 306 controls with 0.6 mg/kg rt-PA. All studies included in this NMA are RCTs, and single-arm studies were not included (S2 Table). The PRISMA Checklist is located in S1 Checklist.

### Risk-of-bias assessment

**Fig 2:**
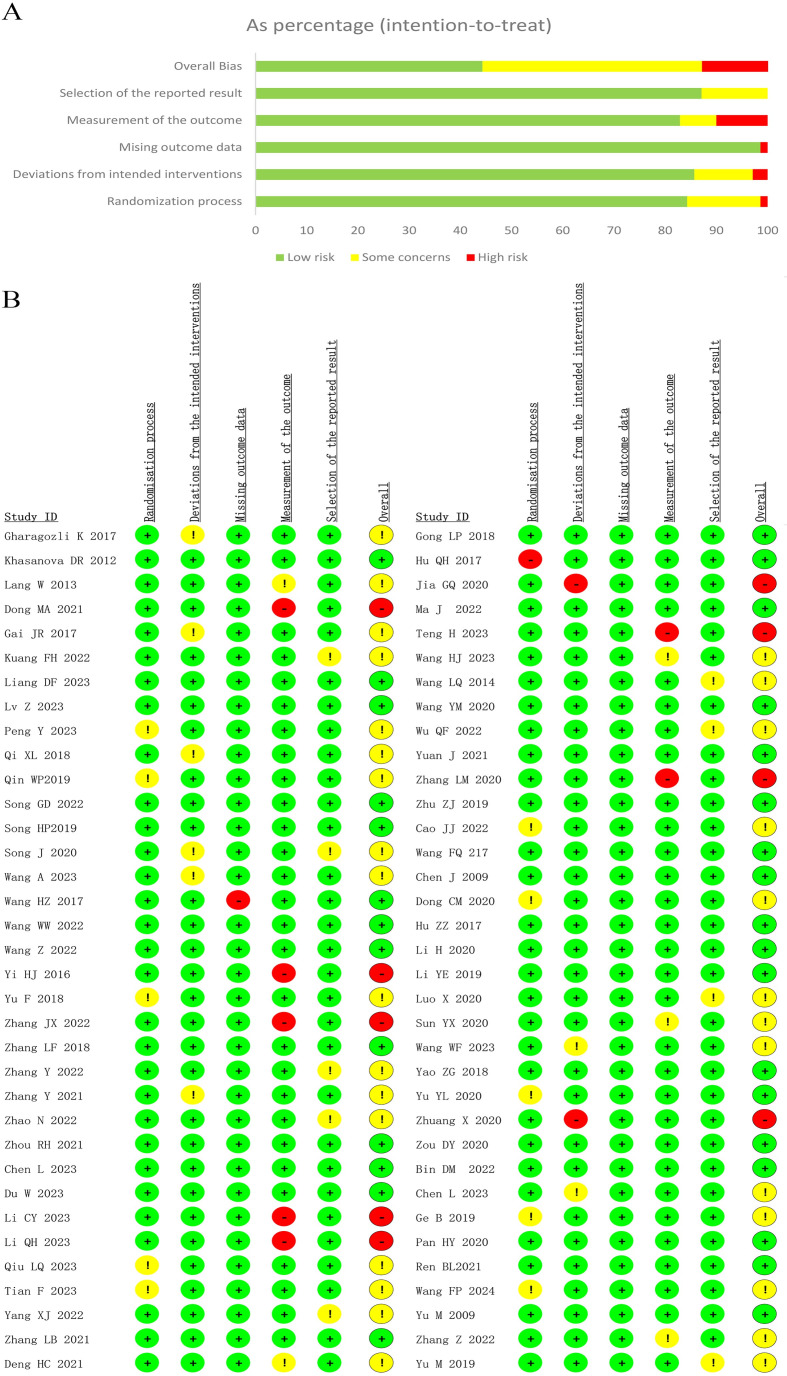
Results for assessing the risk of bias in studies on stroke. (A) Risk of bias graph that displays the overall risk of bias. (B) Risk of bias summary that provides a detailed summary of bias risk in each study.

The Risk of Bias 2.0 Tool was utilized to evaluate the potential bias in all studies included in this review. A significant factor contributing to a high risk of bias was the measurement of outcomes. Overall, 31 trials (44.3%) were judged to have a low risk of bias, 30 trials (42.9%) were identified as having some concerns, and the remaining 9 trials (12.8%) were classified as having a high risk of bias, as illustrated in Fig 2. In summary, the included studies exhibited a generally low to moderate risk of bias.

### Statistical heterogeneity, and consistency assessment, and publication bias

**Fig 3:**
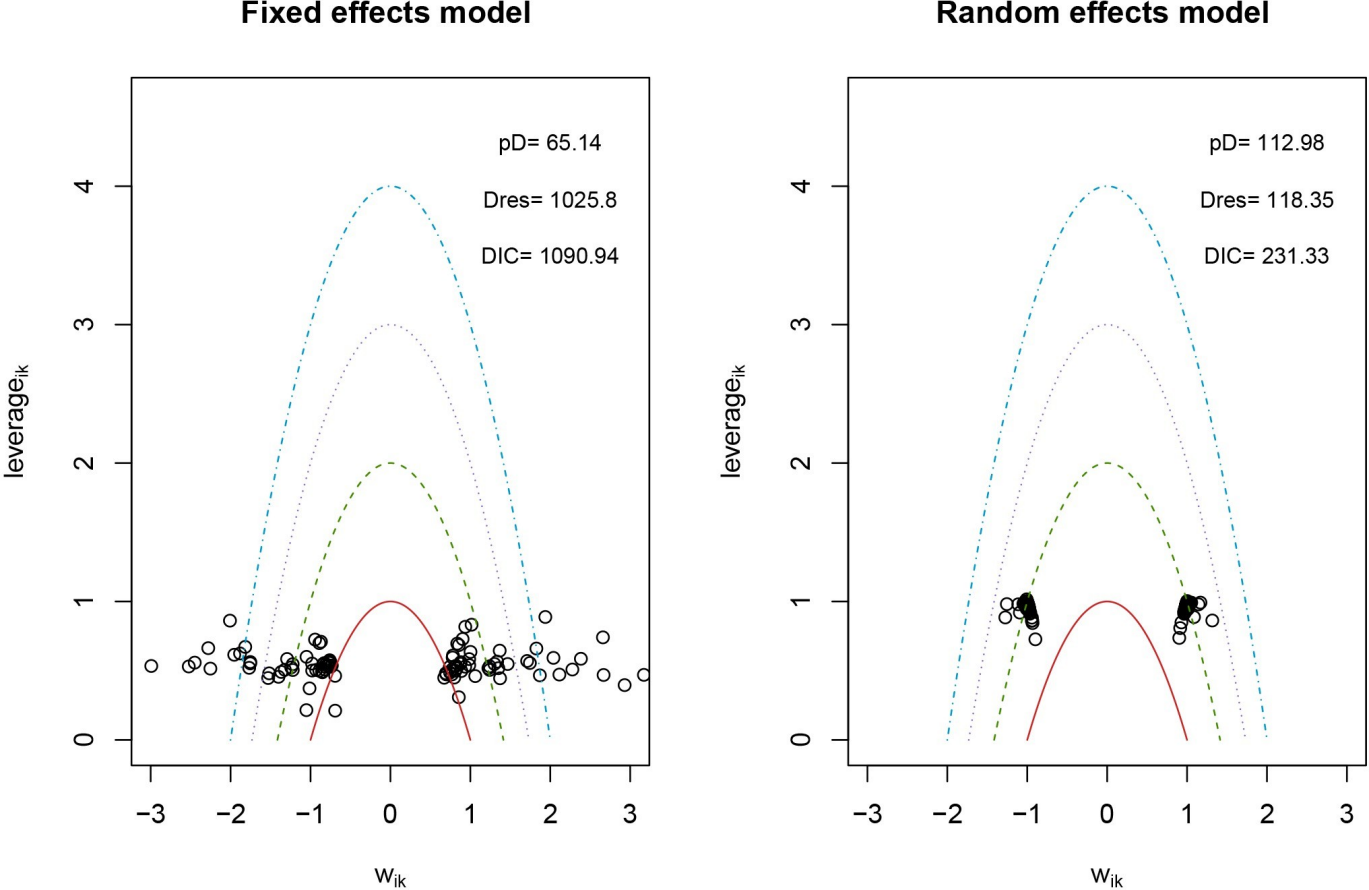
Lever diagram for stroke. The lever diagram represents the comparison between leverageik and Bayesian deviation residuals of all I tests and each of the K arms.

**Fig 4:**
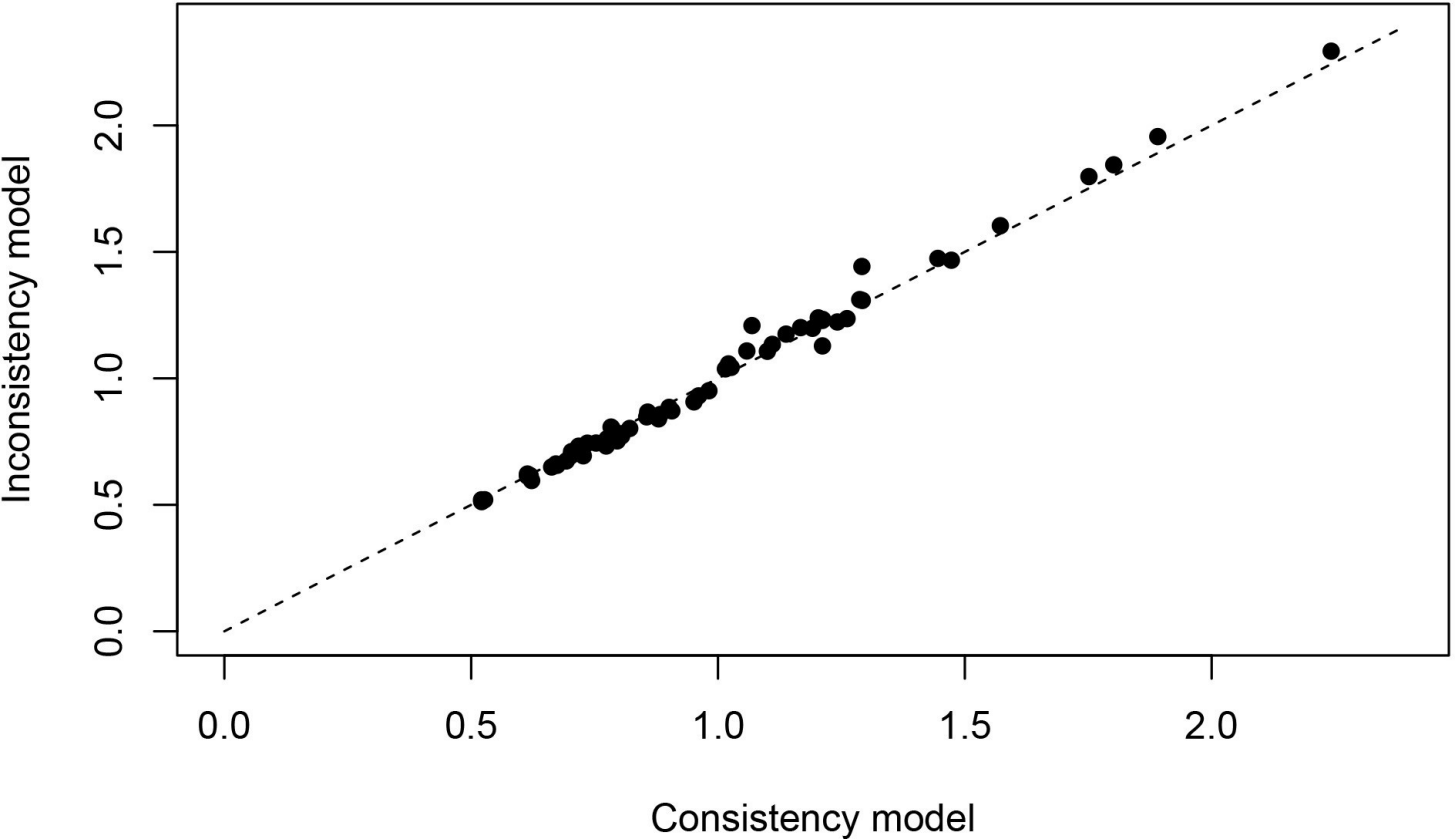
Conformance test for stroke. Conformance test compares the posterior mean deviation of each data group between consistency and the ume m(b) Bias risk evaluation results displayed by including studies odel to judge the consistency among the included studies.

**Fig 5:**
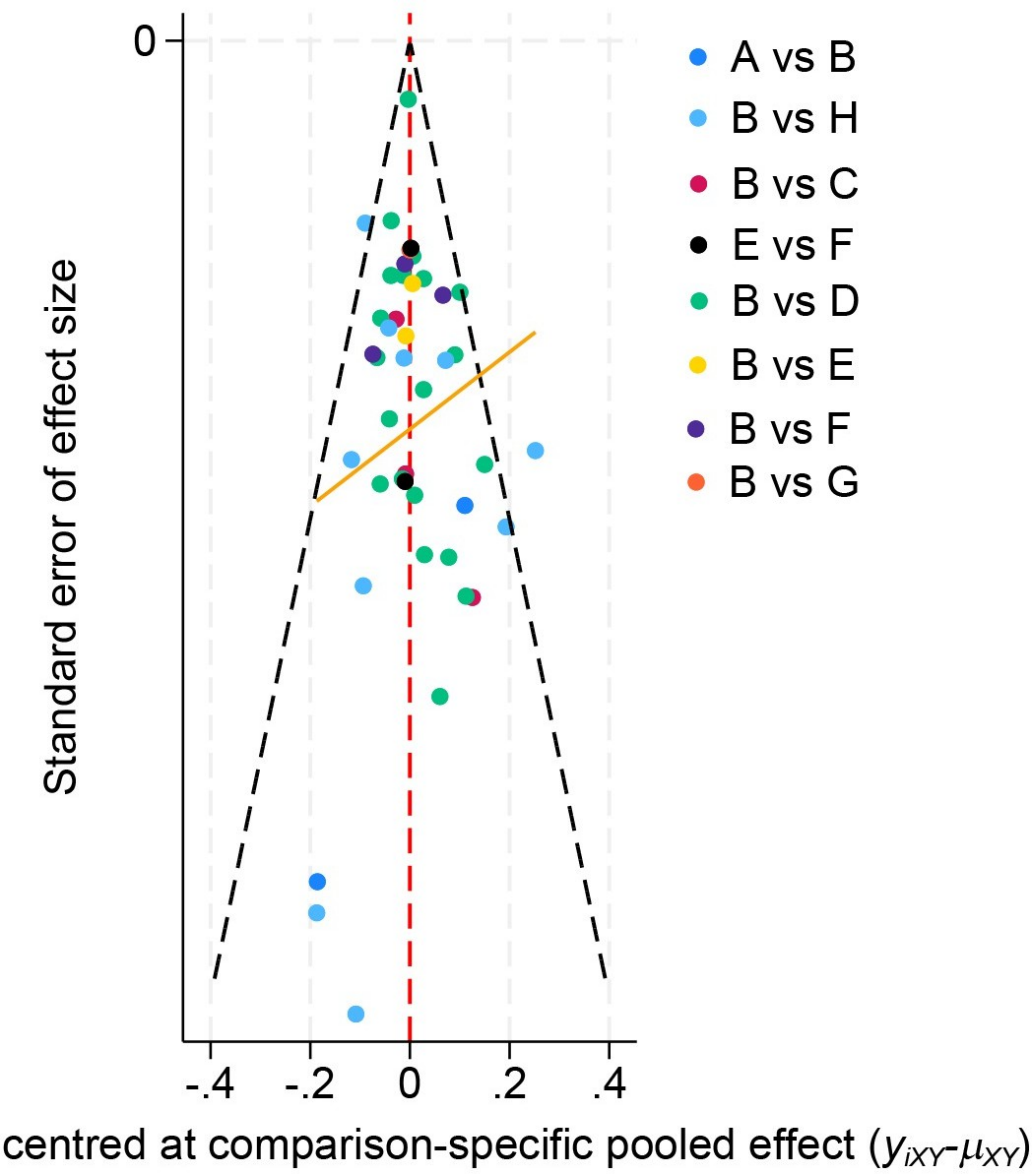
Funnel plots for systematic review and meta-analysis. Symmetry around the central line suggests no publication bias, while asymmetry may indicate potential bias or heterogeneity among studies.

We conducted an analysis to compare the model fit between the fixed-effects model and the random-effects model for each outcome measure, as shown in Fig 3. Our findings revealed that the random-effects model showed a superior fit for each outcome measure. Additionally, we assessed the level of inconsistency in these models by comparing the posterior distribution of the deviance differences between the fit-UME and consistency models. This approach aimed to evaluate the consistency of the results obtained from the included studies, as illustrated in Fig 4. Moreover, the results from the included studies were consistent, thereby supporting the validity of indirect comparisons. To assess publication bias in the included studies, funnel plots were used. These plots, depicted in Fig 5, are symmetrically distributed, suggesting minimal evidence of publication bias.

**Fig 6:**
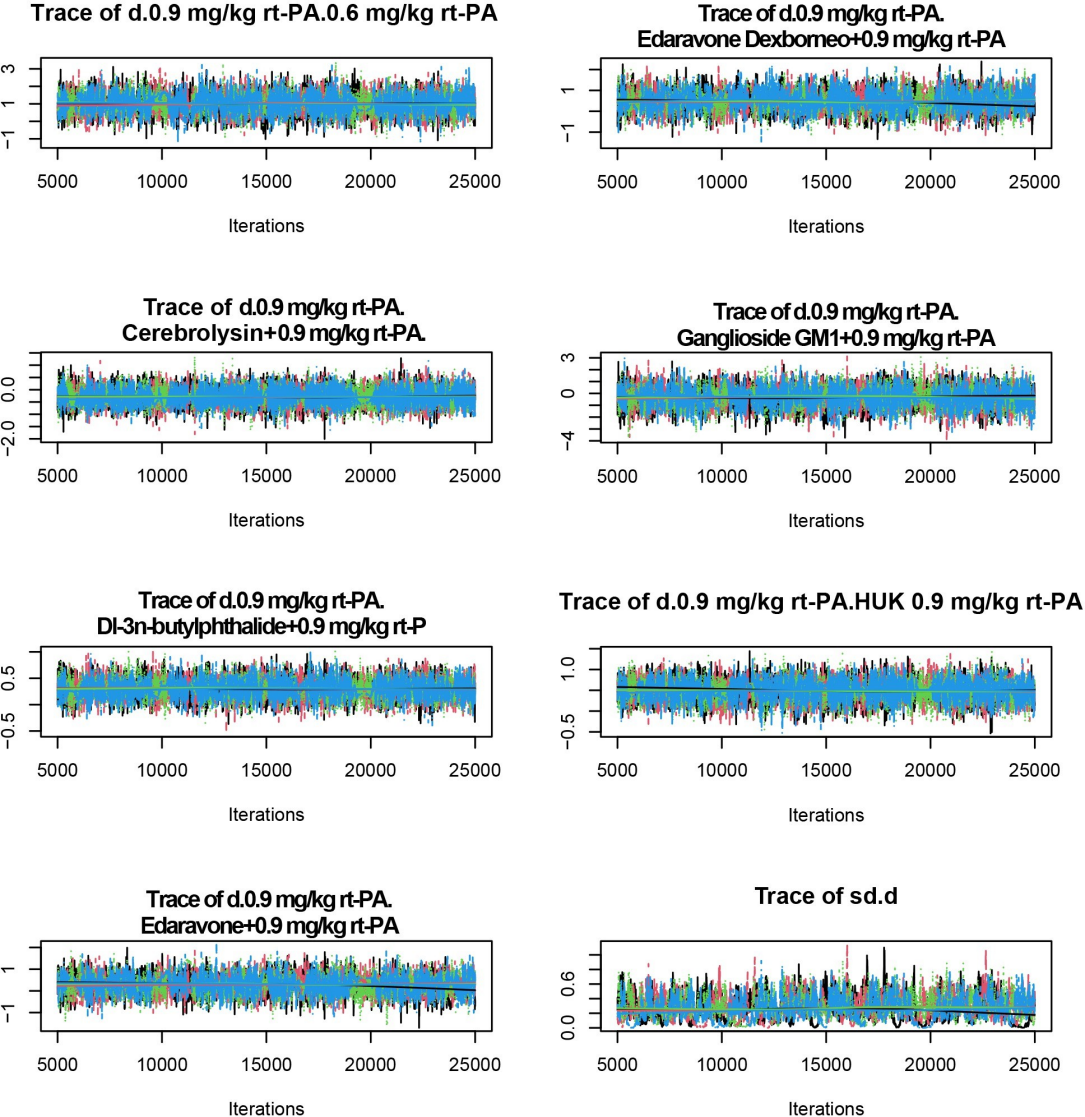
Trajectory plots for assessment of the degree of convergence. The trajectory plots illustrate that the Markov Chain Monte Carlo chain exhibits stable fluctuations and significant overlap once the number of iterations surpasses 5000. (A) NIHSS scores; (B) BI scores; (C) the incidence of adverse reaction. *NIHSS: National Institutes of Health Stroke Scale, BI: Barthel Index.

Trajectory plots graphically represent the evolution of parameter estimates over iterations, allowing us to observe whether the estimates stability over time-a key indicator of model convergence. Each line in the plot represents the trajectory of a parameter estimate across iterations. Convergence suggests the model is reaching a consistent solution. The trajectory plots demonstrate stable fluctuations and significant overlap in the MCMC chain (Fig 6).

**Fig 7:**
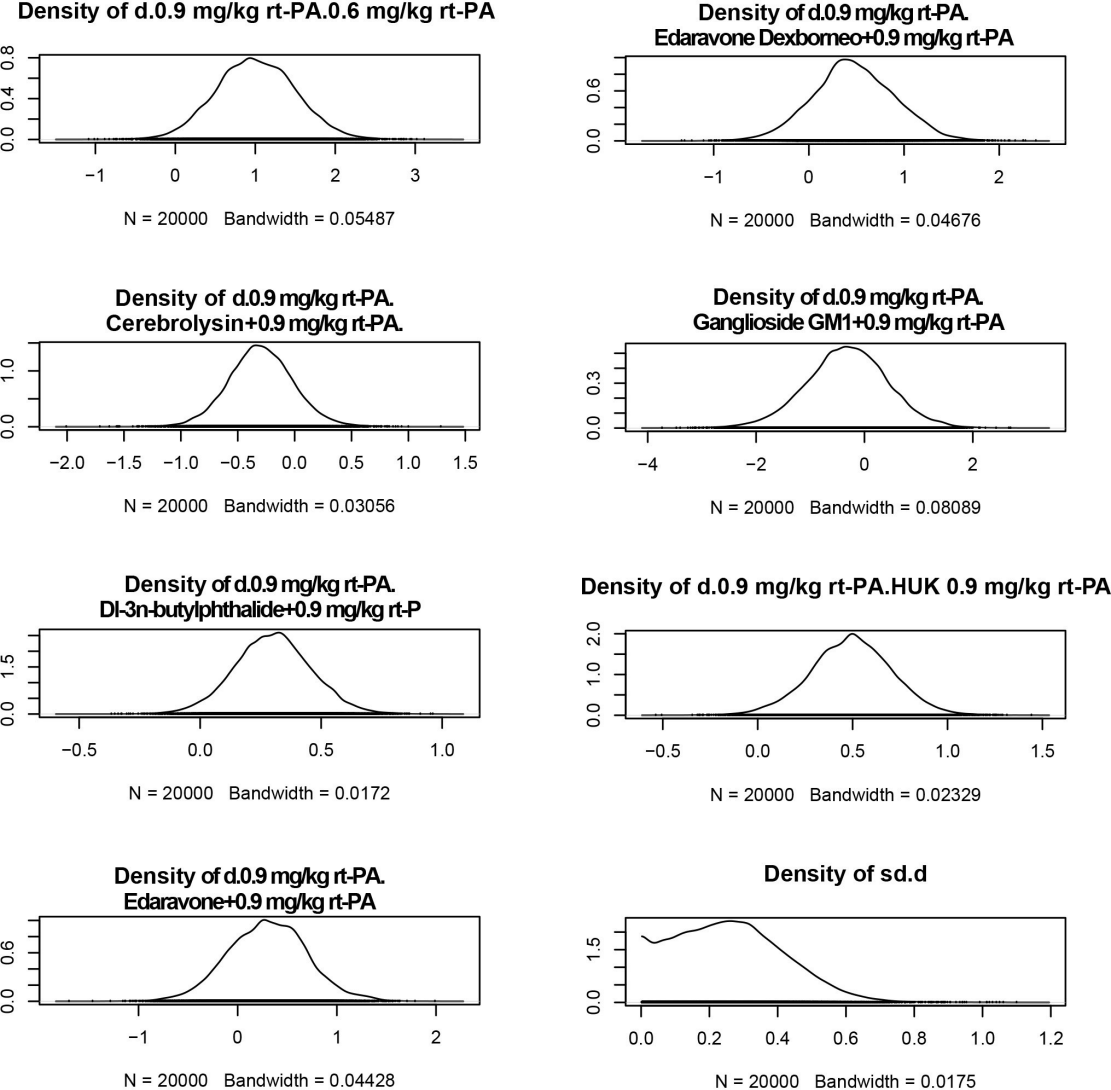
Density map shows stability. The stability of the system is further corroborated by the density map, which illustrates the bandwidth approaching zero and the number of iterations reaching 20,000. Collectively, these observations robustly suggest that the model demonstrates effective convergence (A) NIHSS scores; (B) BI scores; (C) the incidence of adverse reaction. *NIHSS: National Institutes of Health Stroke Scale, BI: Barthel Index.

Meanwhile, density plots provide a visual representation of the distribution of parameter estimates across iterations. Each plot shows the distribution of a parameter estimate. The peak of the curve indicates the most probable value, while the width of the curve reflects the uncertainty or variability of the estimate. Symmetrical plots indicate a well-behaved distribution. These plots allow us to assess the stability and reliability of our model in predicting outcomes in these critical scenarios. The density plots suggest exemplary convergence of the model (Fig 7).

**Fig 8:**
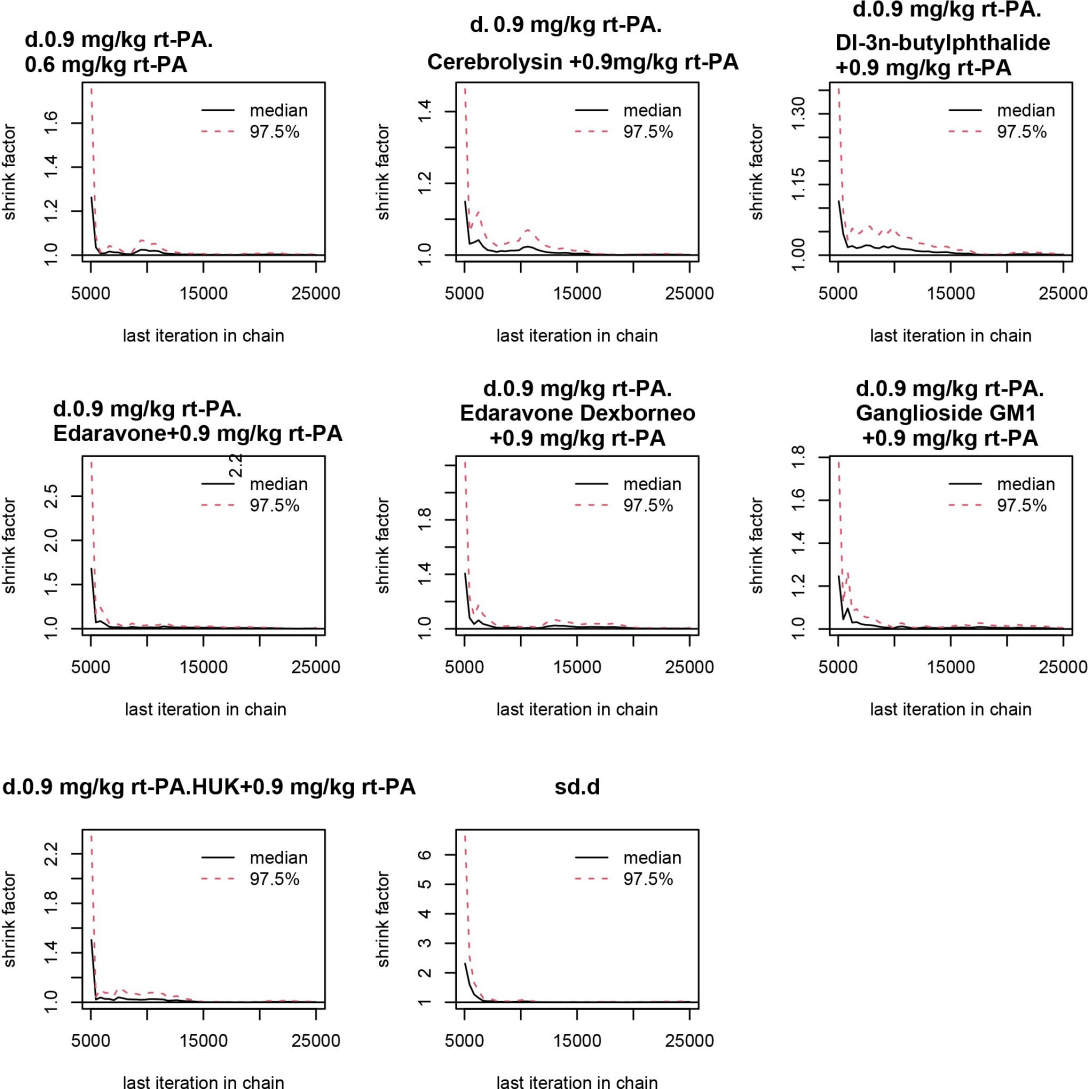
Brooks-Gelman-Rubin diagnostic diagram. When the curve approaches and remains stable around 1, it signifies robust convergence in the Brooks-Gelman-Rubin diagnostic diagram. (A) NIHSS scores; (B) BI scores; (C) adverse effects rate. *NIHSS: National Institutes of Health Stroke Scale, BI: Barthel Index.

In NMA, the Brooks-Gelman-Rubin diagnostic diagram is used to assess the convergence of multiple chains in a model. It compares within-chain and between-chain variances to indicate if more iterations are needed for convergence, with values close to 1 suggesting good convergence. A stable tendency towards 1 in the curve, as depicted in the Brooks-Gelman-Rubin diagnostic diagram, is indicative of robust convergence (Fig 8).

By demonstrating a consistent convergence pattern in the trajectory plots, a stable distribution in the density plots, and the Brooks-Gelman-Rubin diagnostic diagram, the model’s predictions are overall robust.

### Network diagram

In NMA, the network diagram plays a key role in visually representing the evidence structure of the analysis. It maps out all the treatments being compared, and the direct comparisons made between them through studies. Nodes represent different treatments, and connecting lines indicate direct comparisons between these treatments, with the thickness of these lines often reflecting the number of studies or sample size. The size of each node corresponds to the number of studies included in each group. This diagram provides a clear, immediate understanding of the relationships and the volume of evidence available for each treatment comparison, aiding in the assessment of the network’s robustness and completeness.

**Fig 9:**
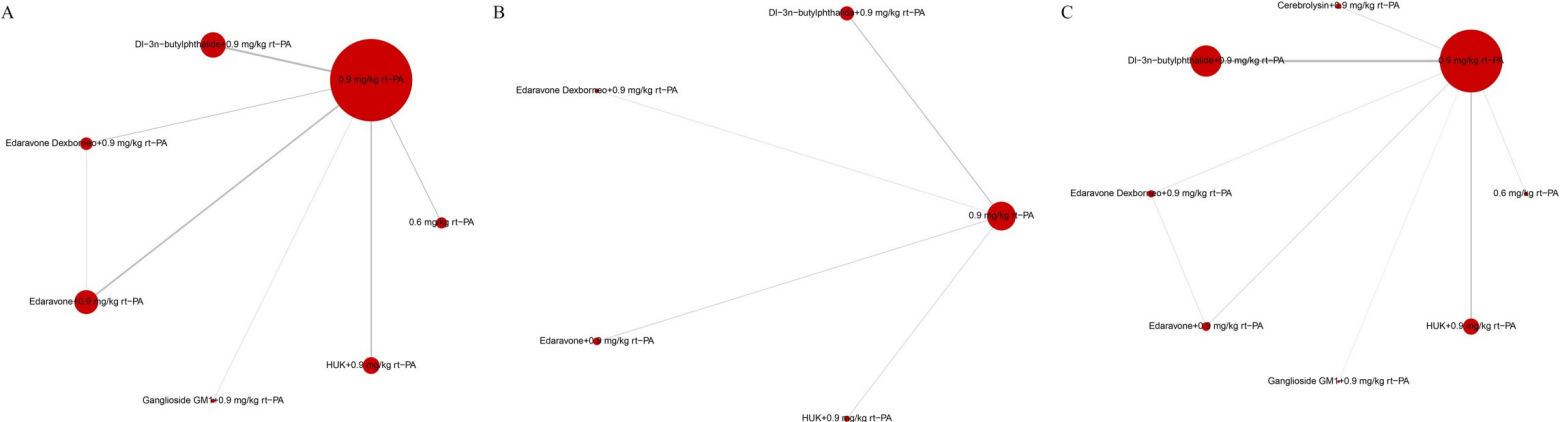
Network meta-analysis diagrams. (A) NIHSS scores; (B) BI scores; (C) the incidence of adverse reaction. *NIHSS: National Institutes of Health Stroke Scale, BI: Barthel Index, HUK: Human urinary kallidinogenase, rt-PA: Recombinant tissue plasminogen activator.

Fig 9 graphically represents the network relationships between various neuroprotective treatments and the control group. The size of each circle in the diagram is proportional to the number of participants receiving that particular intervention. The interventions are denoted by letters, and the thickness of the lines connecting these letters reflects the number of studies comparing those interventions. Notably, 0.9 mg/kg rt-PA was the most extensively studied treatment, followed by Dl-3n-butylphthalide combined with 0.9 mg/kg rt-PA, Edaravone with 0.9 mg/kg rt-PA, and HUK with 0.9 mg/kg rt-PA. The most frequently compared treatment pairs were Dl-3n-butylphthalide with 0.9 mg/kg rt-PA versus 0.9 mg/kg rt-PA, and Edaravone with 0.9 mg/kg rt-PA versus 0.9 mg/kg rt-PA.

### Forest map

In NMA, a forest plot is used to visually summarise the results of pairwise comparisons between different treatments. The function can produce forest plots, which facilitate the comparison of aggregated results of various drugs across different studies and at distinct efficacy levels. This function effectively presents the measures of intervention. In the forest plot of the direct comparison, the different interventions were compared at different efficacy levels.

**Fig 10:**
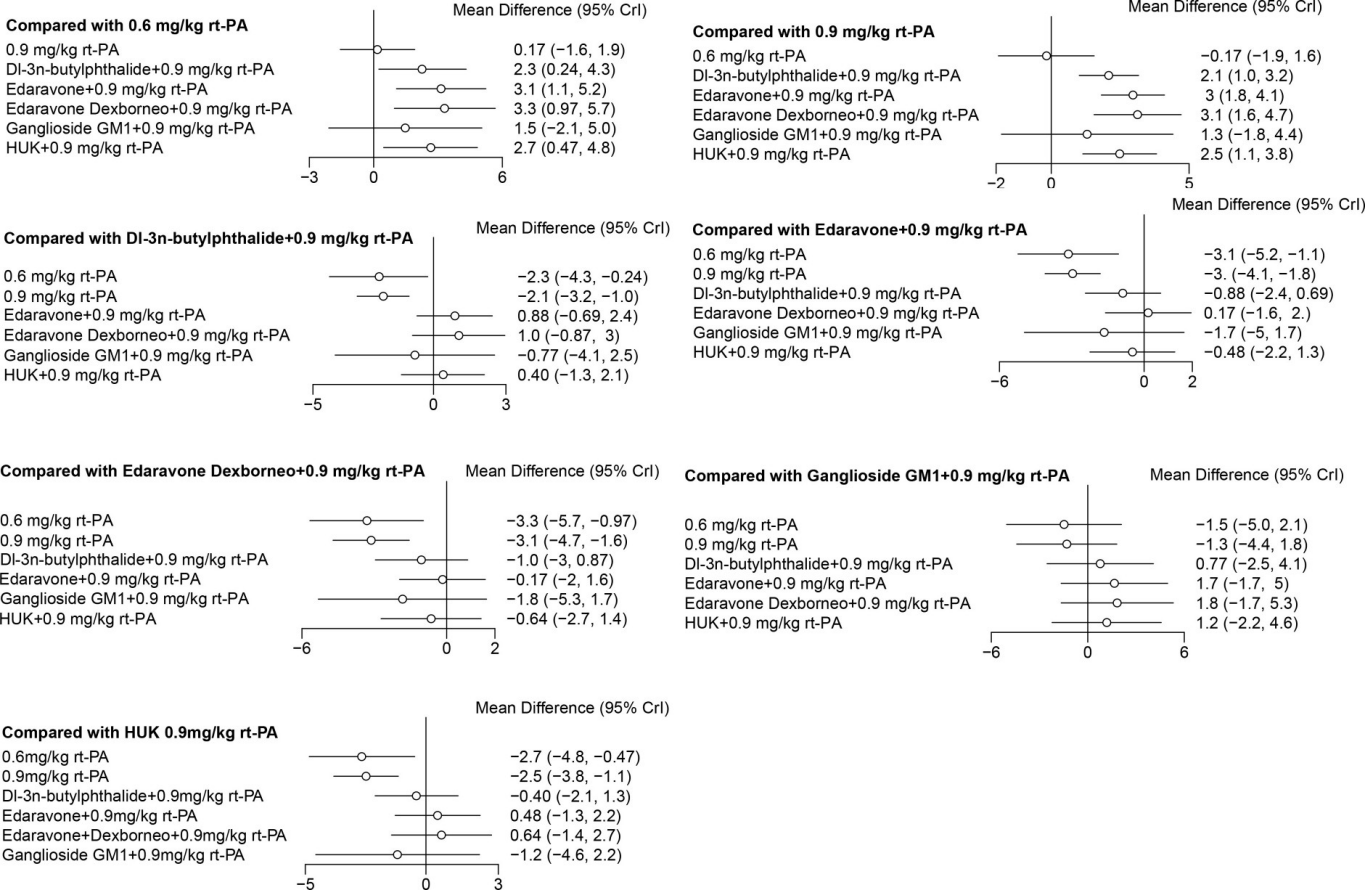
Forest plots represent comprehensive trial data on direct comparison for NIHSS scores of stroke treatments *NIHSS: National Institutes of Health Stroke Scale, rt-PA: Recombinant tissue plasminogen activator.

The treatment efficacy of Dl-3n-butylphthalide with 0.9 mg/kg rt-PA, Edaravone with 0.9 mg/kg rt-PA, Edaravone Dexborneo with 0.9 mg/kg rt-PA, and HUK with 0.9 mg/kg rt-PA was superior to that of both 0.6 mg/kg rt-PA and 0.9 mg/kg rt-PA based on NIHSS scores. However, when compared to 0.6 mg/kg rt-PA, the difference in efficacy with 0.9 mg/kg rt-PA was not statistically significant (Fig 10).

**Fig 11:**
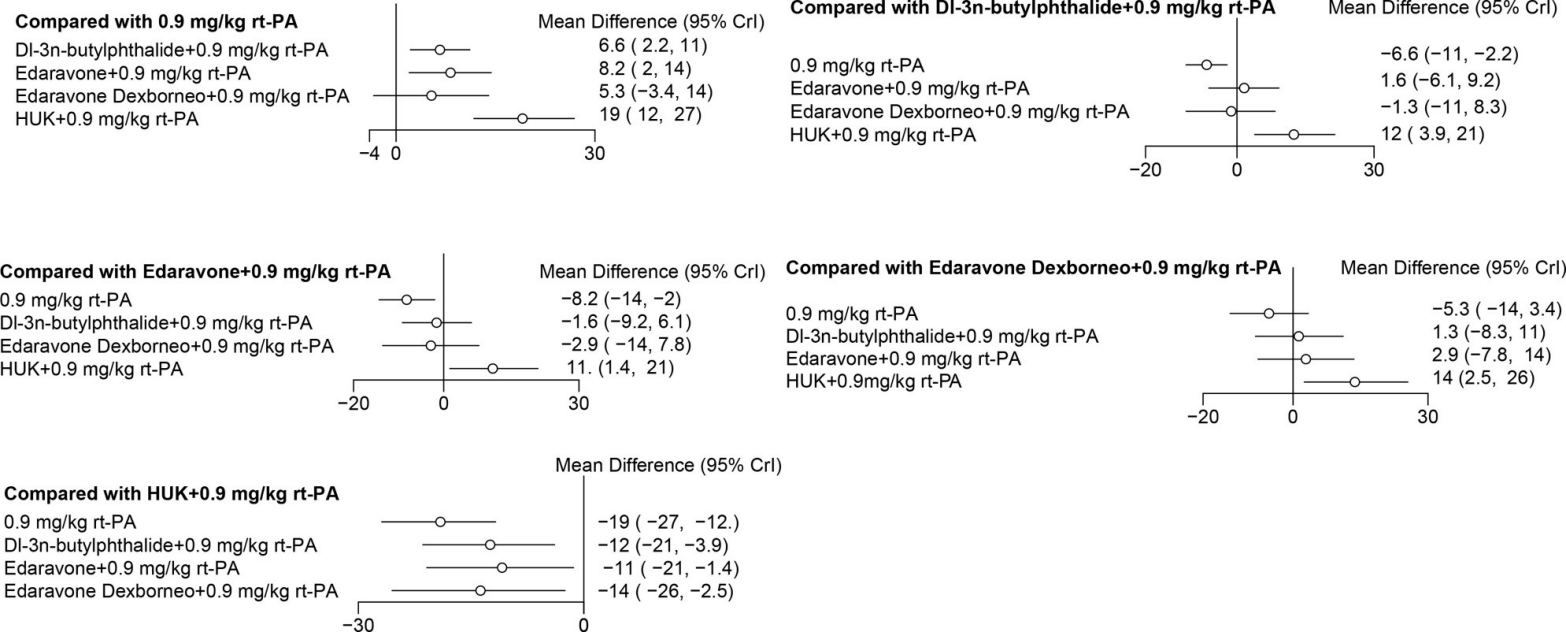
Forest plots represent comprehensive trial data on direct comparison for BI scores of stroke treatments *BI: Barthel Index, rt-PA: Recombinant tissue plasminogen activator.

Dl-3n-butylphthalide with 0.9 mg/kg rt-PA, Edaravone with 0.9 mg/kg rt-PA, and HUK with 0.9 mg/kg rt-PA demonstrated a more pronounced neuroprotective effect than 0.9 mg/kg rt-PA in terms of BI scores (Fig 10B). Among these, HUK with 0.9 mg/kg rt-PA showed superior efficacy on BI scores compared to Dl-3n-butylphthalide with 0.9 mg/kg rt-PA, Edaravone with 0.9 mg/kg rt-PA, and Edaravone Dexborneo with 0.9 mg/kg rt-PA (Fig 11).

**Fig 12:**
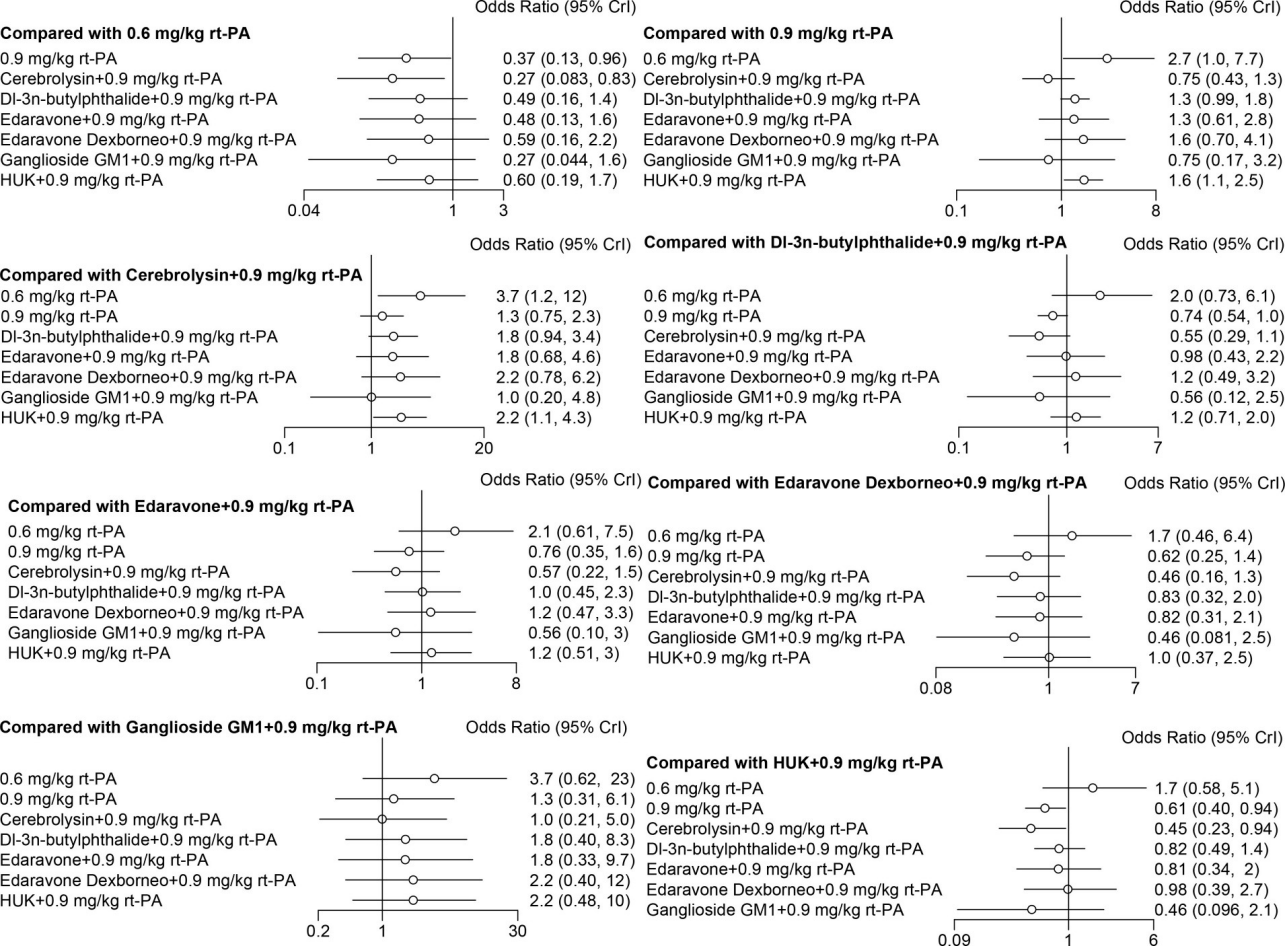
Forest plots represent comprehensive trial data on direct comparison for the incidence of adverse reaction of stroke treatments *rt-PA: Recombinant tissue plasminogen activator.

Regarding the rate of adverse reactions, 0.9 mg/kg rt-PA and Cerebrolysin with 0.9 mg/kg rt-PA presented a higher safety risk compared to 0.6 mg/kg rt-PA. Conversely, HUK with 0.9 mg/kg rt-PA had a lower safety risk than both 0.9 mg/kg rt-PA and Cerebrolysin with 0.9 mg/kg rt-PA. The remaining pairwise comparisons did not reach statistical significance (Fig 12).

### The heatmap of the ranking table

In NMA, the heatmap of the ranking table is a crucial visualization tool. It graphically represents the hierarchy of treatment efficacies or safety profiles by displaying treatments along one axis and their ranking criteria along another. The heatmap generated by the nma.league() function presents a comprehensive estimation of relative effects, allowing for comparisons between any pair of interventions. This heatmap vividly details the ranking of each outcome index, including both the OR and the 95% CI for each outcome index across all intervention groups. This visual representation simplifies the interpretation of complex NMA data, allowing for a more intuitive understanding of which treatment is most effective or safest.

**Fig 13:**
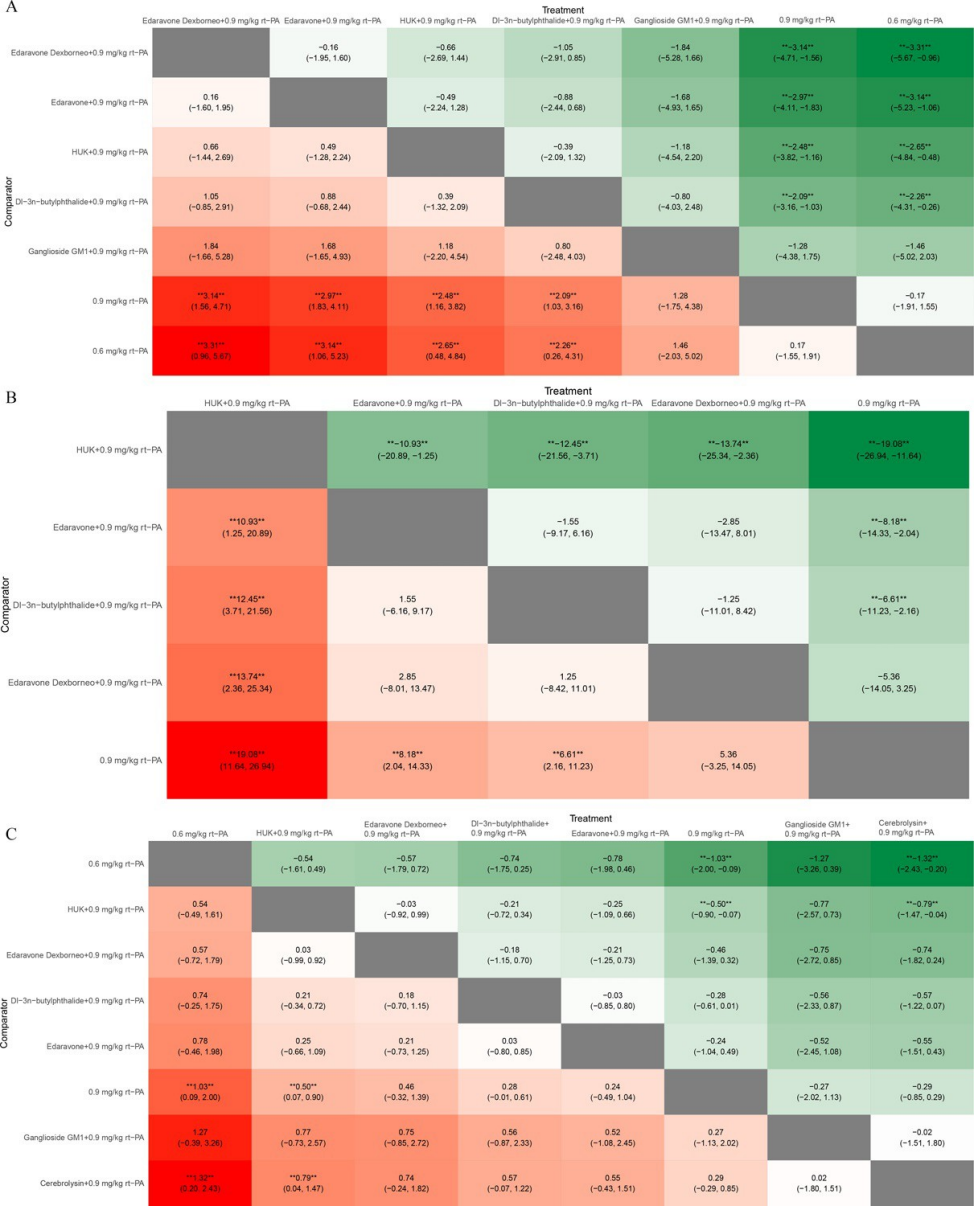
Ranking chart heat map for stroke. This heat map illustrates the relative effect comparisons between various stroke interventions, displaying the odds ratios and 95% confidence intervals for each outcome index across all groups. (A) NIHSS scores ranking chart heat map; (B) BI scores ranking chart heat map; (C) adverse effects rate ranking chart heat map. *NIHSS: National Institutes of Health Stroke Scale, BI: Barthel Index, HUK: Human urinary kallidinogenase, rt-PA: Recombinant tissue plasminogen activator.

Edaravone Dexborneo with 0.9 mg/kg rt-PA, Edaravone with 0.9 mg/kg rt-PA, HUK with 0.9 mg/kg rt-PA, and Dl-3n-butylphthalide with 0.9 mg/kg rt-PA demonstrated significantly greater efficacy compared to both 0.9 mg/kg rt-PA and 0.6 mg/kg rt-PA in NIHSS scores at the 2-week follow-up (Fig 13A). Furthermore, Edaravone with 0.9 mg/kg rt-PA, Dl-3n-butylphthalide with 0.9 mg/kg rt-PA exhibited superior efficacy compared with 0.9 mg/kg rt-PA in BI scores at the 2-week follow-up (Fig 11B). Among these treatments, HUK with 0.9 mg/kg rt-PA exhibited superior efficacy than Edaravone with 0.9 mg/kg rt-PA, Dl-3n-butylphthalide with 0.9 mg/kg rt-PA, Edaravone Dexborneo with 0.9 mg/kg rt-PA, and 0.9 mg/kg rt-PA in terms of BI scores (Fig 13B). In terms of safety, both 0.6 mg/kg rt-PA and HUK with 0.9 mg/kg rt-PA were associated with a lower risk of adverse effects compared to 0.9 mg/kg rt-PA and Cerebrolysin with 0.9 mg/kg rt-PA (Fig 13C).

### SUCRA rankings

SUCRA values, expressed as percentages, provide a clear, quantitative indication of a treatment’s performance relative to others within the network. A higher SUCRA value suggests a better rank, indicating that the treatment is more likely to be among the best options. This method simplifies the interpretation of complex NMA results.

In NMA, a cumulative probability ranking chart is a vital visualization tool. It displays the likelihood of each treatment being ranked at different levels for a specific outcome across all studies included in the analysis. This SUCRA chart helps understanding the overall performance of each treatment in a cumulative manner, considering all available evidence. It presents the probability distribution of rankings, offering a dynamic view of how each treatment compares to others over a range of ranks.

A ranking probability histogram is used to visualize the probability of each treatment being ranked at different positions for a specific outcome. It helps in comprehensively assessing the performance of each treatment relative to others. Each treatment is represented by a bar, with its height indicating the probability of achieving a particular rank.

Based on SUCRA values, various neuroprotective treatments were ranked according to NIHSS scores, BI scores, and adverse reaction rate, as shown in Table 1. The cumulative probability ranking chart, presented in both curves and histogram form, visually displays the ranking probabilities of each treatment.

**Table 1:** SUCRA rankings.

Efficacy				safety	
NIHSS	SUCRA	BI	SUCRA	adverse effects rate	SUCRA
Edaravone Dexborneo+0.9 mg/kg rt-PA	83.82	HUK+0.9 mg/kg rt-PA	99.25	0.6 mg/kg rt-PA	91.12
Edaravone+0.9 mg/kg rt-PA	80.75	Dl-3n-butylphthalide+0.9 mg/kg rt-PA	48.55	HUK+0.9 mg/kg rt-PA	71.24
HUK+0.9 mg/kg rt-PA	66.23	Edaravone Dexborneo+0.9 mg/kg rt-PA	39.71	Edaravone Dexborneo+0.9 mg/kg rt-PA	66.37
Dl-3n-butylphthalide+0.9 mg/kg rt-PA	54.42	Edaravone+0.9 mg/kg rt-PA	39.71	Dl-3n-butylphthalide+0.9 mg/kg rt-PA	54.77
Ganglioside GM1+0.9 mg/kg rt-PA	40.77	0.9 mg/kg rt-PA	2.7	Edaravone+0.9 mg/kg rt-PA	50.74
0.9 mg/kg rt-PA	13.09			0.9 mg/kg rt-PA	27.22
0.6 mg/kg rt-PA	10.94			Ganglioside GM1+0.9 mg/kg rt-PA	25.85
				Cerebrolysin+0.9 mg/kg rt-PA	12.69

*NIHSS: National Institutes of Health Stroke Scale; BI: Barthel Index; SUCRA: surface under the cumulative ranking; HUK: Human urinary kallidinogenase; rt-PA: recombinant tissue plasminogen activator.

The top three treatments based on NIHSS scores at the 2-week follow-up were Edaravone Dexborneo with 0.9 mg/kg rt-PA, Edaravone with 0.9 mg/kg rt-PA, and HUK with 0.9 mg/kg rt-PA. Interestingly, the efficacy of 0.9 mg/kg rt-PA was superior to that of 0.6 mg/kg rt-PA on NIHSS scores, with 0.6 mg/kg rt-PA demonstrating the least effectiveness.

Notably, HUK with 0.9 mg/kg rt-PA, Dl-3n-butylphthalide with 0.9 mg/kg rt-PA, and Edaravone Dexborneo with 0.9 mg/kg rt-PA were the top-ranked treatments for BI scores at the 2-week follow-up, while 0.9 mg/kg rt-PA exhibited the lowest efficacy on BI scores.

**Fig 14:**
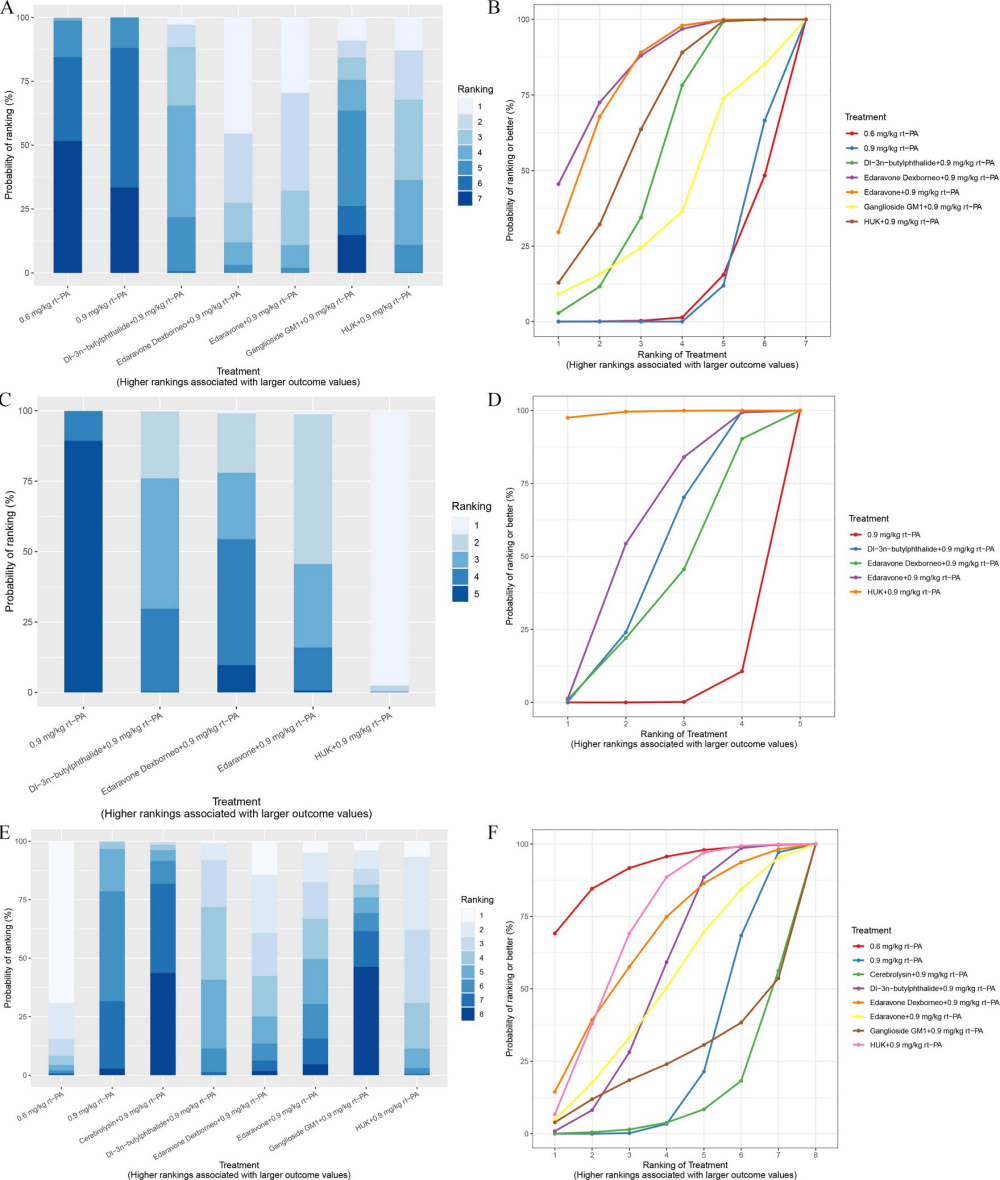
The ranking probability histogram and cumulative probability ranking chart for stroke outcomes are presented. The histogram and SUCRA charts graphically display the ranking probabilities of each intervention group, using bars and curves to illustrate their comparative effectiveness. (A) The histogram chart of NIHSS scores; (B) SUCRA chart of NIHSS scores; (C) The histogram chart of BI scores; (D) SUCRA chart of BI scores; (E) The histogram chart of adverse effects rate; (F) SUCRA chart of adverse effects rate. *SUCRA: surface under the cumulative ranking, NIHSS: National Institutes of Health Stroke Scale, BI: Barthel Index, HUK: Human urinary kallidinogenase, rt-PA: Recombinant tissue plasminogen activator.

SUCRA analysis indicated that the treatments associated with the lowest rates of adverse effects were 0.6 mg/kg rt-PA, HUK with 0.9 mg/kg rt-PA, and Edaravone Dexborneo with 0.9 mg/kg rt-PA, reflecting their excellent safety profiles (Fig 14 and Table 1).

## Discussion

This is the first study to compare neuroprotective agents combined with rt-PA for the treatment of stroke in AIS patients. A Bayesian NMA was conducted to evaluate the efficacy and safety of neuroprotective agents plus rt-PA.

### Principal findings

This NMA of 70 RCTs, encompassing 4,140 patients with AIS using different neuroprotective strategies and 4,012 controls, provides a comprehensive evaluation of various neuroprotective strategies in addition to rt-PA. The study’s robust methodology, exclusively incorporating RCTs, lends credibility to its findings, and the large sample size enhances the generalisability of the results.

The broad range of neuroprotective treatments analyzed, including 0.6 mg/kg rt-PA, 0.9 mg/kg rt-PA, HUK+0.9 mg/kg rt-PA, Edaravone Dexborneo+0.9 mg/kg rt-PA, Dl-3n-butylphthalide+0.9 mg/kg rt-PA, Edaravone+0.9 mg/kg rt-PA, Ganglioside GM1+0.9 mg/kg rt-PA, Cerebrolysin+0.9 mg/kg rt-PA, reflects the diversity of therapeutic options available for AIS. The variation in the number of RCTs and patient populations for each intervention underscores the need for a thorough and nuanced understanding of each treatment’s efficacy and safety. Notably, the NIHSS and BI scores served as critical measures of treatment effectiveness. The efficacy was evaluated in short-term follow-up (2 weeks). These scores are pivotal in assessing the severity of neurological deficits of stroke patients. The safety profile, a critical aspect of treatment evaluation, was highlighted through the analysis of adverse effects rate. This assessment is paramount in clinical decision-making, especially when considering the risk-benefit balance of neuroprotective strategies.

These findings suggest that Edaravone Dexborneo combined with 0.9 mg/kg rt-PA, Edaravone with 0.9 mg/kg rt-PA, HUK with 0.9 mg/kg rt-PA, and Dl-3n-butylphthalide with 0.9 mg/kg rt-PA demonstrate significant neuroprotective effects in the short-term follow-up (2 weeks) based on NIHSS and BI scores. The treatments associated with the lowest incidence of adverse effects were 0.6 mg/kg rt-PA, HUK with 0.9 mg/kg rt-PA, and Edaravone Dexborneo with 0.9 mg/kg rt-PA, highlighting their excellent safety profiles. Controversies regarding the efficacy and safety of this non-standard dose (0.6 mg/kg rt-PA) continue to exist (28959472). Notably, in terms of NIHSS scores, the efficacy of the 0.6 mg/kg rt-PA was the least favorable; however, its safety profile was superior. While SUCRA rankings indicated that the efficacy of 0.9 mg/kg rt-PA was better than that of 0.6 mg/kg rt-PA based on NIHSS scores, direct comparisons in the Forest plot showed no statistically significant difference between the two dosages. Conversely, the Forest plot comparison demonstrated a statistically significant higher safety for 0.6 mg/kg rt-PA compared to 0.9 mg/kg rt-PA. (36457872, 33814371, 32248771). In this study, the low-dose group (0.6 mg/kg) exhibited no difference in effectiveness but showed better safety compared to the standard-dose group (0.9 mg/kg).

Edaravone (3-methyl-1-phenyl-2-pyrazolin-5-one) is a lipophilic antioxidant capable of crossing the blood-brain barrier. Edaravone’s neuroprotective mechanisms include scavenging reactive oxygen and nitrogen species, enhancing antioxidant enzyme activity, and safeguarding neurons, glial cells, and vascular cells from oxidative stress [95]. This medication received official approval for AIS in Japan in 2001 and has since been extensively utilized in several Asian countries, including China and India [96–98]. Edaravone Dexborneol, a novel antioxidant and anti-inflammatory neuroprotectant, is composed of edaravone and (+)-borneol in a 4:1 ratio that restrains inflammatory processes, oxidative pathways, and caspase-related apoptosis pathways [99]. Pharmacological studies on the efficacy of edaravone combined with (+)-borneol have demonstrated that Edaravone Dexborneol exhibits a synergistic effect and provides a longer treatment duration compared to edaravone alone. This indicates that Edaravone Dexborneol offers superior protection against stroke compared to the marketed edaravone [100–102]. In this study, Edaravone Dexborneol+rt-PA showed better efficacy than Edaravone+ rt-PA on NIHSS scores and. The safety profile of Edaravone Dexborneol combined with rt-PA was superior to that of edaravone combined with rt-PA. Additionally, both short- and long-term economic analyses suggest that edaravone dexborneol is highly likely to be a cost-effective alternative for treating AIS compared to edaravone, dl-3-n-butylphthalide, and HUK in China. [101, 103, 104]. Additionally, dl-3n-butylphthalide, a synthesised compound, represents a novel therapeutic agent independently developed in China for the treatment of acute cerebral infarction. This innovative drug targets multiple pathological pathways involved in AIS. Its neuroprotective properties include attenuating platelet aggregation, enhancing mitochondrial function, reducing neurovascular inflammation, and protecting neuronal cells from ischemia-induced and neurotoxic damage [105]. Furthermore, Dl-3n-butylphthalide improves cerebral blood flow, reduces brain oedema, and preserves the integrity of the blood-brain barrier [106, 107]. In addition, HUK, a glycoprotein extracted from human urine, regulates the kallikrein-kinin system, activates bradykinin B1 and B2 receptors, and promotes cerebral perfusion and post-ischemic angiogenesis [108]. HUK has been approved by the China Food and Drug Administration for stroke treatment for more than a decade [109]. Notably, pharmacological evidence has proven that the use of rt-PA plus HUK on acute cerebral infarction can significantly improve neurological deficit, long-term prognosis, and quality of life. This may be related to the biological effects of inhibition of urinary kallikrein on ischemia-reperfusion inflammation, expansion of the tiny blood vessels, improvement of circulation, and inhibition of apoptosis [110–112]. In this study, the combination of rt-PA with HUK significantly reduced NIHSS and BI scores. HUK+rt-PA exhibited the lowest incidence of adverse reactions. Moreover, cerebrolysin, a neuropeptide, has neurotrophic properties by reducing free radical generation, inhibiting neuroinflammation, improving brain cellular survival, and stimulating neurogenesis [113]. Cerebrolysin has a therapeutic effect on tPA- and fibrin-induced impairment of cerebral endothelial cell permeability by reducing proinflammatory factors and increasing the levels of tight junction proteins [114]. Ganglioside GM1, a member of the ganglioside family, is a promising alternative treatment for stroke. The pathophysiological mechanisms of neuroprotection for Ganglioside GM1 include decreasing the expression of NMDA receptor, reducing the content of mitochondrial calcium, and increasing the expression of glutamate and aspartate in neurons [115]. In summary, compared to rt-PA alone, the combination treatments of Edaravone+rt-PA, Edaravone Dexborneol+rt-PA, HUK+rt-PA, Dl-3n-butylphthalide+rt-PA, and Ganglioside GM1+rt-PA have shown superior efficacy. Neuroprotective agents such as Edaravone, Edaravone Dexborneol, HUK, Dl-3n-butylphthalide, and Ganglioside GM1 can serve as effective adjuncts to rt-PA therapy. Among these, Edaravone Dexborneol+rt-PA not only demonstrates significant efficacy and safety but also exhibits excellent cost-effectiveness, positioning it as a highly economical and effective treatment option.

### Strengths

The efficacy of single neuroprotective agents in treating AIS remains controversial. However, in this study, all neuroprotective agents combined with rt-PA demonstrated superior efficacy compared to rt-PA alone. This finding suggests that combining neuroprotective agents with rt-PA represents a promising therapeutic approach. Additionally, we opted for the standard dose to mitigate the influence of varying rt-PA dosages. According to the American Heart Association/American Stroke Association guidelines, the standard dose (0.9 mg/kg) is the optimal selection for AIS [116].

This NMA allows for the simultaneous comparison of multiple neuroprotective interventions in addition to rt-PA, even when some have not been directly compared in head-to-head trials. This provides a more comprehensive overview of the efficacy and safety of available neuroprotective treatments. By synthesizing data from multiple studies, NMA makes efficient use of existing data, which is especially valuable when primary data collection is costly or infeasible. NMA can rank interventions based on their effectiveness, providing valuable insights for clinicians and policymakers on the most effective interventions. NMA incorporates both direct and indirect comparisons, increasing the statistical power and the ability to draw conclusions where direct comparison data is limited.

### Limitations

There were several limitations in this study. Firstly, NIHSS scores and BI scores were used to assess the efficacy of stroke treatments at 2 weeks, lacking long-term follow-ups. Some studies were excluded due to insufficient clinical trials. Secondly, many studies included in this analysis had small sample sizes. In NMA, although the automatic weighting by statistical software can balance the impact of studies with varying sample sizes to some extent, it does not completely eliminate the potential influence of disparities in patient numbers. Ongoing RCTs testing the efficacy of multiple neuroprotective treatments in addition to rt-PA in AIS patients are hopeful in providing larger sample sizes and improving the certainty of evidence for clinical translation. Thirdly, most of the studies included in this NMA were from Asian countries, with a few from Europe and the United States, and none from Africa. The overrepresentation of Asian studies may lead to regional bias, affecting the overall conclusions and global applicability of the findings. Fourthly, since all studies included in this research are RCTs, there was no adjustment for covariates in the original studies, such as age, sex, and dosage. Through randomization and blinding, it can be assumed that patients in both the intervention and control groups are homogenous regarding confounding factors other than the intervention. Nonetheless, heterogeneity between studies is inevitable. We tested for inter-study heterogeneity and used a random-effects model for analyses with significant heterogeneity. Finally, while subgroup or sensitivity analyses are common methods to address heterogeneity, our study is limited by a small number of direct comparison studies and constraints of indirect comparison methods, preventing in-depth subgroup and sensitivity analyses.

## Conclusion

In summary, this is the first review of neuroprotective strategies for the treatment of stroke using Bayesian NMA. This comprehensive NMA reveals the relative effectiveness and safety of various neuroprotective agents in addition to rt-PA for AIS. Combining rt-PA with neuroprotective agents such as Edaravone, Edaravone Dexborneol, HUK, Dl-3n-butylphthalide, and Ganglioside GM1 shows significant clinical benefits compared to rt-PA alone. These combinations enhance neuroprotection, reduce brain damage, improve functional recovery, and lower adverse reaction rates. This suggests that these combination therapies can offer better outcomes for patients with AIS. These findings support the potential integration of these combination therapies into standard AIS treatment, aiming for improved patient outcomes and personalized therapeutic approaches.

## Supporting information

S1 Table The specific search strategy. The detailed search strategy used for the meta-analysis, including the databases searched, keywords, and terms applied, as well as any filters or limits set on the search.
(DOCX)

S2 Table Basic characteristics of the included studies. The basic characteristics including the authors, publication year, sample size, study design, interventions, type of control group, primary outcome measures, and their definitions.
(PDF)

S1 Checklist PRISMA checklist. (DOCX)
