# Supplementary material for: Synergistic effects of neuroprotective drugs with intravenous recombinant tissue plasminogen activator in acute ischemic stroke: A Bayesian network meta-analysis
Source: PLoS One. 2024 Dec 2;19(12):e0311231. doi: 10.1371/journal.pone.0311231 (PMC11611160; doi:10.1371/journal.pone.0311231)
Supplement: S1 Table — The detailed search strategy used for the meta-analysis, including the databases searched, keywords, and terms applied, as well as any filters or limits set on the search. (DOCX) [file pone.0311231.s001.docx]

**The specific search strategy**

PubMed

((Stroke[MeSH Terms] OR Brain Ischemia[MeSH Terms] OR Cerebral Infarction[MeSH Terms]) AND (Cerebrolysin[MeSH Terms] OR cerebrolysine[tiab] OR Citicoline[MeSH Terms] OR Butylphthalide[MeSH Terms] OR DL-3-n-butylphthalide[MeSH Terms] OR 3-n-butylphthalide[tiab] OR l-3-n-butylphthalide[tiab] OR NBP[tiab] OR Magnesium[MeSH Terms] OR Magnesium Sulfate[MeSH Terms] OR Minocycline[MeSH Terms] OR human urinary kallidinogenase[tiab] OR HUK[tiab] OR Edaravone[MeSH Terms] OR Intracranial Thrombosis[MeSH Terms] OR MCI-186[tiab] OR norphenazone[tiab] OR Edaravone dexborneol[tiab] OR Eda-Dex[tiab] OR Edaravone and Dexborneol[tiab] OR Vinpocetine [MeSH Terms] OR Cavinton[tiab] OR Gangliosides [MeSH Terms] AND GM1 [Subheading] OR Gangliosides [MeSH Terms] AND Ganglioside-monosialic acid [Subheading] OR Neuroprotective Agents [MeSH Terms] OR FAST-MAG[tiab] OR tPA[tiab] OR rTPA[tiab] OR Reperfusion Therapy[tiab] OR alteplase[tiab] OR hypothermia[tiab] OR EVT[tiab] OR endovascular treatment[tiab] OR NA-1[tiab] OR Nerinetide[tiab] OR uric acid[tiab] OR immunomodulators[tiab] OR neuroprotectant therapy[tiab] OR thrombolysis[tiab]) AND (Recovery of Function[MeSH Terms] OR Mortality[MeSH Terms]))

EMBASE

('cerebrovascular accident'/exp OR 'brain ischemia'/exp OR 'brain infarction'/exp) AND ('cerebrolysin'/exp OR 'citicoline'/exp OR 'butylphthalide'/exp OR 'magnesium'/exp OR 'minocycline'/exp OR 'human urinary kallidinogenase'/exp OR 'edaravone'/exp OR 'vinpocetine'/exp OR 'ganglioside'/exp OR 'neuroprotective agent'/exp OR 'edaravone dexborneol' OR 'FAST-MAG' OR 'tPA' OR 'rTPA' OR 'Reperfusion Therapy' OR 'alteplase' OR 'hypothermia' OR 'endovascular treatment' OR 'NA-1' OR 'Nerinetide' OR 'uric acid' OR 'immunomodulators' OR 'neuroprotectant therapy' OR 'thrombolysis') AND ('convalescence'/exp OR 'mortality rate'/exp)

Cochrane Library

#1 (ti,ab,kw.Stroke OR ti,ab,kw.Brain Ischemia OR ti,ab,kw.Cerebral Infarction)

#2 (ti,ab,kw.Cerebrolysin OR ti,ab,kw.crebrolysine OR ti,ab,kw.Citicoline OR

ti,ab,kw.Butylphthalide OR ti,ab,kw.DL-3-n-butylphthalide OR ti,ab,kw.3-n-butylphthalide* OR ti,ab,kw.l-3-n-butylphthalide* OR ti,ab,kw.NBP OR ti,ab,kw.Magnesium OR ti,ab,kw.MgSO4 OR ti,ab,kw.Magnesium Sulfate OR ti,ab,kw.Minocycline OR ti,ab,kw.human urinary kallidinogenase OR ti,ab,kw.HUK OR ti,ab,kw.Edaravone OR ti,ab,kw.Intracranial Thrombosis* OR ti,ab,kw.MCI-186 OR ti,ab,kw.norphenazone OR ti,ab,kw.Edaravone dexborneol* OR

ti,ab,kw.Eda-Dex* OR ti,ab,kw.Edaravone and Dexborneol* OR ti,ab,kw.Vinpocetine OR

ti,ab,kw.Cavinton OR ti,ab,kw.GM1* OR ti,ab,kw.Gangliosides Ganglioside-monosialic acid* OR ti,ab,kw.Neuroprotective Agents OR ti,ab,kw.FAST-MAG OR ti,ab,kw.tPA OR ti,ab,kw.rTPA OR ti,ab,kw.Reperfusion Therapy OR ti,ab,kw.alteplase OR ti,ab,kw.hypothermia OR ti,ab,kw.EVT OR ti,ab,kw.endovascular treatment OR ti,ab,kw.NA-1 OR ti,ab,kw.Nerinetide OR ti,ab,kw.uric acid OR ti,ab,kw.immunomodulators OR ti,ab,kw.neuroprotectant therapy OR ti,ab,kw.thrombolysis)

#3 (ti,ab,kw.Recovery of Function* OR Mortality)

#4 #1 AND #2 AND #3

CNKI

(胞磷胆碱 OR 脑活素 OR 施普善OR脑活素 OR 丁苯酞 OR 神经节苷脂 OR 依达拉奉 OR 依达拉奉右莰醇 OR 先必新 OR 长春西汀 OR 镁 OR 米诺环素 OR 人尿激肽原酶 OR 脑保护剂 OR 神经保护剂) AND (脑卒中 OR 脑梗 OR 缺血性脑卒中) AND (神经保护 OR 低温治疗 OR 阿替普酶 OR 溶栓 OR 免疫调节剂 OR 尿酸)

Wanfang Data

(“胞磷胆碱” OR “脑活素” OR “施普善” OR“脑活素”OR “丁苯酞” OR “神经节苷脂” OR “依达拉奉” OR “依达拉奉右莰醇” OR “先必新” OR “长春西汀” OR “镁” OR “米诺环素” OR “人尿激肽原酶” OR “脑保护剂” OR “神经保护剂”) AND (“脑卒中” OR “脑梗” OR “缺血性脑卒中”) AND (“神经保护” OR “低温治疗” OR “阿替普酶” OR “溶栓” OR “免疫调节剂” OR “尿酸”)

ProQuest

("Citicoline" OR "Cerebrolysin" OR "Butylphthalide" OR "Gangliosides" OR "Edaravone" OR "Edaravone Dexborneol" OR "Vinpocetine" OR "Magnesium" OR "Minocycline" OR "Human Urinary Kallidinogenase" OR "Neuroprotective Agents" OR "Stroke" OR "Brain Ischemia" OR "Cerebral Infarction" OR "Neuroprotection" OR "Hypothermia Treatment" OR "Alteplase" OR "Thrombolysis" OR "Interventional Surgery" OR "Immunomodulators" OR "Uric Acid") AND ("Recovery of Function" OR "Mortality")

Open Grey

"Stroke" AND ("Citicoline" OR "Cerebrolysin" OR "Butylphthalide" OR "Gangliosides" OR "Edaravone" OR "Neuroprotective Agents" OR "Thrombolysis" OR "Hypothermia Treatment")

Google Scholar

("Citicoline" OR "Cerebrolysin" OR "Butylphthalide" OR "Gangliosides" OR "Edaravone" OR "Edaravone Dexborneol" OR "Vinpocetine" OR "Magnesium" OR "Minocycline" OR "Human Urinary Kallidinogenase" OR "Neuroprotective Agents" OR "FAST-MAG" OR "tPA" OR "rTPA" OR "Reperfusion Therapy" OR "Alteplase" OR "Hypothermia" OR "Endovascular Treatment" OR "NA-1" OR "Nerinetide" OR "Uric Acid" OR "Immunomodulators" OR "Neuroprotectant Therapy") AND("Stroke" OR "Brain Ischemia" OR "Cerebral Infarction") AND ("Recovery of Function" OR "Mortality")
