# Supplementary material for: Synergistic effects of neuroprotective drugs with intravenous recombinant tissue plasminogen activator in acute ischemic stroke: A Bayesian network meta-analysis
Source: PLoS One. 2024 Dec 2;19(12):e0311231. doi: 10.1371/journal.pone.0311231 (PMC11611160; doi:10.1371/journal.pone.0311231)
Supplement: S2 Table — The basic characteristics including the authors, publication year, sample size, study design, interventions, type of control group, primary outcome measures, and their definitions. (PDF) [file pone.0311231.s002.pdf]

S2 Table 2 Basic characteristics of the included studies

First author	Publication year	Patients of IG (M:F)		Patients of CG(M:F)		Age of IG, CG(mean±	Age of CG, CG(mean±SD)	Interventions therapy	Interventions dose, route	Control therapy	Control measures, dose, route	Follow-up	Outcome indicators
Gharagozli K	2017	50	(27:23)	50	(26:24)	NA	NA	Cerebrolysin+rt-PA	30ml, iv, qd	rt-PA	rt-PA 0.9 mg/kg, iv,qd	2 weeks	③
Khasanova DR	2012	126	NA	117	NA	55.0-77.8	55.0-70.5	Cerebrolysin+rt-PA	30ml, iv, qd	rt-PA	rt-PA 0.9 mg/kg, iv,qd	2 weeks	③
Lang W	2013	60	NA	59	NA	65.5	67	Cerebrolysin+rt-PA	30ml, iv, qd	rt-PA	rt-PA 0.9 mg/kg, iv,qd	2 weeks	③
Dong MA	2021	39	(18:21)	36	(17:19)	65.44±5.15	69.95±5.07	NBP+rt-PA	NBP 25mg, iv, bid; rt-PA 0.9 mg/kg, iv,qd	rt-PA	rt-PA 0.9 mg/kg, iv,qd	3months	①②③
Gai JR	2017	54	(28:26)	54	(24:30)	63.94±4.85	65.81±5.36	NBP+rt-PA	NBP 25mg, iv, bid; rt-PA 0.9 mg/kg, iv,qd	rt-PA	rt-PA 0.9 mg/kg, iv,qd	2 weeks	①②③
Kuang FH	2022	28	(16:12)	28	(17:11)	61.32±4.61	61.24±4.57	NBP+rt-PA	NBP 25mg, iv, bid; rt-PA 0.9 mg/kg, iv,qd	rt-PA	rt-PA 0.9 mg/kg, iv,qd	2 weeks	①②③
Liang DF	2023	26	(20:6)	26	(20:6)	58.88±3.03	58.92±3.01	NBP+rt-PA	NBP 25mg, iv, bid; rt-PA 0.9 mg/kg, iv,qd	rt-PA	rt-PA 0.9 mg/kg, iv,qd	2 weeks	①②③
Lv Z	2023	51	(33:18)	51	(35:16)	65.82±5.14	64.48±4.97	NBP+rt-PA	NBP 25mg, iv, bid; rt-PA 0.9 mg/kg, iv,qd	rt-PA	rt-PA 0.9 mg/kg, iv,qd	2 weeks	①③
Peng Y	2023	25	(12:13)	25	(11:14)	59.67±5.36	60.17±5.68	NBP+rt-PA	NBP 25mg, iv, bid; rt-PA 0.9 mg/kg, iv,qd	rt-PA	rt-PA 0.9 mg/kg, iv,qd	2 weeks	①③
Qi XL	2018	56	(31:25)	56	(36:20)	60.8±4.8	5.6±4.3	NBP+rt-PA	NBP 25mg, iv, bid; rt-PA 0.9 mg/kg, iv,qd	rt-PA	rt-PA 0.9 mg/kg, iv,qd	3months	①③
Qin WP	2019	30	(21:9)	31	(21:10)	62.17±8.52	61355±8.82	NBP+rt-PA	NBP 25mg, iv, bid; rt-PA 0.9 mg/kg, iv,qd	rt-PA	rt-PA 0.9 mg/kg, iv,qd	2 weeks	①②③
Song GD	2022	47	(20:27)	47	(19:28)	68.05±2.21	67.72±2.09	NBP+rt-PA	NBP 25mg, iv, bid; rt-PA 0.9 mg/kg, iv,qd	rt-PA	rt-PA 0.9 mg/kg, iv,qd	2 weeks	①②
Song HP	2019	47	(24:23)	47	(25:22)	61.20±7.26	61.68±7.53	NBP+rt-PA	NBP 25mg, iv, bid; rt-PA 0.9 mg/kg, iv,qd	rt-PA	rt-PA 0.9 mg/kg, iv,qd	2 weeks	①③
Song J	2020	46	(24:22)	46	(25:21)	62.43±3.41	62.29±3.55	NBP+rt-PA	NBP 25mg, iv, bid; rt-PA 0.9 mg/kg, iv,qd	rt-PA	rt-PA 0.9 mg/kg, iv,qd	2 weeks	①②
Wang A	2023	607	(412:195)	609	(415:194)	56-72	57-74	NBP+rt-PA	NBP 25mg, iv, bid; rt-PA 0.9 mg/kg, iv,qd	rt-PA	rt-PA 0.9 mg/kg, iv,qd	3months	③
Wang HZ	2017	50	(29:21)	50	(27:23)	58.22±11.87	57.57±11.27	NBP+rt-PA	NBP 25mg, iv, bid; rt-PA 0.9 mg/kg, iv,qd	rt-PA	rt-PA 0.9 mg/kg, iv,qd	2 weeks	②③
Wang WW	2022	44	(25:19)	44	(26:18)	58.64±6.38	58.16±6.24	NBP+rt-PA	NBP 25mg, iv, bid; rt-PA 0.9 mg/kg, iv,qd	rt-PA	rt-PA 0.9 mg/kg, iv,qd	2 weeks	①③
Wang Z	2022	50	(26:24)	50	(25:25)	63.22±4.06	62.13±5.46	NBP+rt-PA	NBP 25mg, iv, bid; rt-PA 0.9 mg/kg, iv,qd	rt-PA	rt-PA 0.9 mg/kg, iv,qd	2 weeks	①③
Yu F	2018	50	(32:18)	50	(33:17)	50.56±5.25	52.45±5.77	NBP+rt-PA	NBP 25mg, iv, bid; rt-PA 0.9 mg/kg, iv,qd	rt-PA	rt-PA 0.9 mg/kg, iv,qd	2 weeks	①②③
Zhang JX	2022	51	(30:21)	50	(28:22)	62.19±4.33	61.42±4.25	NBP+rt-PA	NBP 25mg, iv, bid; rt-PA 0.9 mg/kg, iv,qd	rt-PA	rt-PA 0.9 mg/kg, iv,qd	2 weeks	①②③
Zhang LF	2018	58	(30:28)	58	(32:26)	66.6±7.2	66.1±7.8	NBP+rt-PA	NBP 25mg, iv, bid; rt-PA 0.9 mg/kg, iv,qd	rt-PA	rt-PA 0.9 mg/kg, iv,qd	2 weeks	①③
Zhang Y	2022	49	(29:20)	49	(27:22)	56.67±8.25	58.15±9.34	NBP+rt-PA	NBP 25mg, iv, bid; rt-PA 0.9 mg/kg, iv,qd	rt-PA	rt-PA 0.9 mg/kg, iv,qd	2 weeks	①②
Zhang Y	2021	53	(28:25)	53	(27:26)	59.71±16.24	59.42±15.63	NBP+rt-PA	NBP 25mg, iv, bid; rt-PA 0.9 mg/kg, iv,qd	rt-PA	rt-PA 0.9 mg/kg, iv,qd	2 weeks	①②
Zhao N	2022	46	(26:20)	46	(24:22)	70.11±4.59	69.12±3.46	NBP+rt-PA	NBP 25mg, iv, bid; rt-PA 0.9 mg/kg, iv,qd	rt-PA	rt-PA 0.9 mg/kg, iv,qd	2 weeks	①③
Zhou RH	2021	50	(21:29)	50	(22:28)	64.4±11.5	63.6±11.4	NBP+rt-PA	NBP 25mg, iv, bid; rt-PA 0.9 mg/kg, iv,qd	rt-PA	rt-PA 0.9 mg/kg, iv,qd	2 weeks	①③
Chen L	2023	63	(39:24)	63	(41:22)	65.63±3.92	64.76±3.69	EDB+rt-PA	EDB 15ml, iv, bid; rt-PA 0.9 mg/kg, iv,qd	rt-PA	rt-PA 0.9 mg/kg, iv,qd	2 weeks	①②③
Du W	2023	39	(15:24)	38	(14:24)	52.47±5.58	53.09±5.41	EDB+rt-PA	EDB 15ml, iv, bid; rt-PA 0.9 mg/kg, iv,qd	rt-PA	rt-PA 0.9 mg/kg, iv,qd	2 weeks	①②
Li CY	2023	62	NA	62	NA	NA	NA	EDB+rt-PA	EDB 15ml, iv, bid; rt-PA 0.9 mg/kg, iv,qd	rt-PA	rt-PA 0.9 mg/kg, iv,qd	2 weeks	①③
Li QH	2023	50	(35:15)	50	(36:14)	56.63±5.02	58.71±6.24	EDB+rt-PA	EDB 15ml, iv, bid; rt-PA 0.9 mg/kg, iv,qd	rt-PA	rt-PA 0.9 mg/kg, iv,qd	2 weeks	①③
Qiu LQ	2023	30	(17:13)	30	(16:14)	70.0±3.2	69.7±3.6	EDB+rt-PA	EDB 15ml, iv, bid; rt-PA 0.9 mg/kg, iv,qd	rt-PA	rt-PA 0.9 mg/kg, iv,qd	2 weeks	①③
Tian F	2023	53	(26:27)	52	(24:28)	64.18±3.41	63.35±3.26	EDB+rt-PA	EDB 15ml, iv, bid; rt-PA 0.9 mg/kg, iv,qd	rt-PA	rt-PA 0.9 mg/kg, iv,qd	2 weeks	①②
Yang XJ	2022	83	(47:36)	67	(38:29)	70.02±3.14	69.83±3.67	EDB+rt-PA	EDB 15ml, iv, bid; rt-PA 0.9 mg/kg, iv,qd	rt-PA	rt-PA 0.9 mg/kg, iv,qd	2 weeks	①③
Zhang LB	2021	40	(22:18)	40	(19:21)	62.5±3.8	59.5±3.7	EDB+rt-PA	EDB 15ml, iv, bid; rt-PA 0.9 mg/kg, iv,qd	rt-PA	rt-PA 0.9 mg/kg, iv,qd	2 weeks	①②③
Deng HC	2021	45	(24:21)	45	(26:19)	67.34±4.40	65.32±4.55	Edaravone+rt-PA	Edaravone 30mg, iv, bid; rt-PA 0.9 mg/kg, iv,qd	rt-PA	rt-PA 0.9 mg/kg, iv,qd	2 weeks	①
Gong LP	2018	30	(17:13)	30	(16:14)	58.06±10.37	57.88±10.51	Edaravone+rt-PA	Edaravone 30mg, iv, bid; rt-PA 0.9 mg/kg, iv,qd	rt-PA	rt-PA 0.9 mg/kg, iv,qd	2 weeks	①②
Hu QH	2017	23	(11:12)	26	(13:13)	65.8±4.2	65.6±3.8	Edaravone+rt-PA	Edaravone 30mg, iv, bid; rt-PA 0.9 mg/kg, iv,qd	rt-PA	rt-PA 0.9 mg/kg, iv,qd	2 weeks	①
Jia GQ	2020	49	(25:24)	49	(27:22)	61.48±6.89	63.08±6.16	Edaravone+rt-PA	Edaravone 30mg, iv, bid; rt-PA 0.9 mg/kg, iv,qd	rt-PA	rt-PA 0.9 mg/kg, iv,qd	2 weeks	①②
Ma J	2022	54	(32:22)	54	(30:24)	64.12±5.67	64.33±5.29	Edaravone+rt-PA	Edaravone 30mg, iv, bid; rt-PA 0.9 mg/kg, iv,qd	rt-PA	rt-PA 0.9 mg/kg, iv,qd	2 weeks	①
Teng H	2023	60	(35:25)	60	(33:27)	58.21±7.54	68.37±5.71	Edaravone+rt-PA	Edaravone 30mg, iv, bid; rt-PA 0.9 mg/kg, iv,qd	rt-PA	rt-PA 0.9 mg/kg, iv,qd	2 weeks	①
Wang HJ	2023	46	(28:18)	46	(26:20)	62.76±7.35	62.12±7.14	Edaravone+rt-PA	Edaravone 30mg, iv, bid; rt-PA 0.9 mg/kg, iv,qd	rt-PA	rt-PA 0.9 mg/kg, iv,qd	2 weeks	①②
Wang LQ	2014	35	(20:15)	35	(21:14)	65.5±4.7	67.3±5.2	Edaravone+rt-PA	Edaravone 30mg, iv, bid; rt-PA 0.9 mg/kg, iv,qd	rt-PA	rt-PA 0.9 mg/kg, iv,qd	2 weeks	①
Wang YM	2020	48	(27:21)	48	(26:22)	59.21±6.18	58.33±6.21	Edaravone+rt-PA	Edaravone 30mg, iv, bid; rt-PA 0.9 mg/kg, iv,qd	rt-PA	rt-PA 0.9 mg/kg, iv,qd	2 weeks	①②
Wu QF	2022	50	(27:23)	50	(30:20)	56-79	54-79	Edaravone+rt-PA	Edaravone 30mg, iv, bid; rt-PA 0.9 mg/kg, iv,qd	rt-PA	rt-PA 0.9 mg/kg, iv,qd	2 weeks	①②③
Yi HJ	2016	58	(29:29)	57	（30：27）	NA	NA	Edaravone+rt-PA	Edaravone 30mg, iv, bid; rt-PA 0.9 mg/kg, iv,qd	rt-PA	rt-PA 0.9 mg/kg, iv,qd	2 weeks	①
Yuan J	2021	50	(30:20)	50	(28:22)	64.76±6.75	64.45±6.86	Edaravone+rt-PA	Edaravone 30mg, iv, bid; rt-PA 0.9 mg/kg, iv,qd	rt-PA	rt-PA 0.9 mg/kg, iv,qd	2 weeks	①
Zhang LM	2020	38	(20:18)	38	(19:19)	63.15±6.96	64.25±6.72	Edaravone+rt-PA	Edaravone 30mg, iv, bid; rt-PA 0.9 mg/kg, iv,qd	rt-PA	rt-PA 0.9 mg/kg, iv,qd	2 weeks	①
Zhu ZJ	2019	23	(13:10)	27	(15:12)	68.34±2.41	70.04±2.03	Edaravone+rt-PA	Edaravone 30mg, iv, bid; rt-PA 0.9 mg/kg, iv,qd	rt-PA	rt-PA 0.9 mg/kg, iv,qd	2 weeks	①
Cao JJ	2022	78	(46:32)	68	(38:30)	55.09±5.31	54.86±5.36	Ganglioside GM1+rt-	Ganglioside GM 60mg, iv, qd; rt-PA0.9 mg·kg, iv, qd	rt-PA	rt-PA 0.9 mg/kg, iv,qd	2 weeks	①③
Wang FQ	30	78	(17:13)	30	(16:14)	52.87±9.66	53.08±8.09	Ganglioside GM1+rt-	Ganglioside GM 40mg, iv, qd; rt-PA0.6 mg/kg, iv, qd	rt-PA	rt-PA 0.9 mg/kg, iv,qd	2 weeks	①
Chen J	2009	22	NA	22	NA	NA	NA	HUK+rt-PA	HUK0.15PNAU/d, iv,qd; rt-PA 0.9 mg/kg, iv,qd	rt-PA	rt-PA 0.9 mg/kg, iv,qd	3 months	②③
Dong CM	2020	57	(33:24)	57	(29:28)	55.37±5.11	54.97±5.05	HUK+rt-PA	HUK0.15PNAU/d, iv,qd; rt-PA 0.9 mg/kg, iv,qd	rt-PA	rt-PA 0.9 mg/kg, iv,qd	3 months	①②③
Ge B	2019	88	(43:45)	88	(47:41)	58.1±5.5	58.4±4.9	HUK+rt-PA	HUK0.10PNAU/d, iv,qd; rt-PA 0.9 mg/kg, iv,qd	rt-PA	rt-PA 0.9 mg/kg, iv,qd	2 weeks	①③
Hu ZZ	2017	80	(45:35)	80	(50:30)	58.23±10.14	56.47±9.69	HUK+rt-PA	HUK0.15PNAU/d, iv,qd; rt-PA 0.9 mg/kg, iv,qd	rt-PA	rt-PA 0.9 mg/kg, iv,qd	2 weeks	①②
Li H	2020	43	40-73	43	40-72	54.61±5.96	55.25±6.01	HUK+rt-PA	HUK0.15PNAU/d, iv,qd; rt-PA 0.9 mg/kg, iv,qd	rt-PA	rt-PA 0.9 mg/kg, iv,qd	2 weeks	①③
Li YE	2019	44	(24:20)	44	(23:21)	58.48±2.39	58.45±2.36	HUK+rt-PA	HUK0.15PNAU/d, iv,qd; rt-PA 0.9 mg/kg, iv,qd	rt-PA	rt-PA 0.9 mg/kg, iv,qd	2 weeks	①③
Luo X	2020	67	(41:26)	67	(38:29)	61.04±7.18	62.44±7.37	HUK+rt-PA	HUK0.15PNAU/d, iv,qd; rt-PA 0.9 mg/kg, iv,qd	rt-PA	rt-PA 0.9 mg/kg, iv,qd	2 weeks	①②

Sun YX	2020	50	(26:24)	50	(25:25)	67.32±7.11	66.43±6.84	HUK+rt-PA	HUK0.15PNAU/d, iv,qd; rt-PA 0.9 mg/kg, iv,qd	rt-PA	rt-PA 0.9 mg/kg, iv,qd	2 weeks	①③
Wang WF	2023	42	(27:15)	42	(20:22)	65.98±4.82	66.09±4.23	HUK+rt-PA	HUK0.15PNAU/d, iv,qd; rt-PA 0.9 mg/kg, iv,qd	rt-PA	rt-PA 0.9 mg/kg, iv,qd	2 weeks	①②③
Yao ZG	2018	200	(113:87)	200	(111:89)	61.93±12.45	62.15±12.37	HUK+rt-PA	HUK0.15PNAU/d, iv,qd; rt-PA 0.9 mg/kg, iv,qd	rt-PA	rt-PA 0.9 mg/kg, iv,qd	2 weeks	①②
Yu YL	2020	36	(24:12)	36	(21:15)	64.23±3.16	64.45±3.27	HUK+rt-PA	HUK0.15PNAU/d, iv,qd; rt-PA 0.9 mg/kg, iv,qd	rt-PA	rt-PA 0.9 mg/kg, iv,qd	2 weeks	①③
Zhuang X	2020	40	(21:19)	40	(22:18)	64.44±3.10	64.24±3.22	HUK+rt-PA	HUK0.15PNAU/d, iv,qd; rt-PA 0.9 mg/kg, iv,qd	rt-PA	rt-PA 0.9 mg/kg, iv,qd	2 weeks	①③
Zou DY	2020	40	(23:17)	40	(21:19)	67.81±6.24	66.98±5.84	HUK+rt-PA	HUK0.15PNAU/d, iv,qd; rt-PA 0.9 mg/kg, iv,qd	rt-PA	rt-PA 0.9 mg/kg, iv,qd	2 weeks	①③
Bin DM	2022	40	(25:15)	40	(26:14)	82.12±1.23	81.98±1.26	rt-PA	rt-PA 0.9 mg/kg, iv,qd	rt-PA	rt-PA 0.6 mg/kg, iv,qd	2 weeks	③
Chen L	2023	100	(70:30)	51	(31:20)	63.13±12.23	67.45±14.40	rt-PA	rt-PA 0.9 mg/kg, iv,qd	rt-PA	rt-PA 0.6 mg/kg, iv,qd	2 weeks	①
Pan HY	2020	46	(28:18)	46	(29:17)	67.93±1.24	67.34±1.18	rt-PA	rt-PA 0.9 mg/kg, iv,qd	rt-PA	rt-PA 0.6 mg/kg, iv,qd	2 weeks	①
Ren BL	2021	30	(19:11)	30	(18:12)	59.6±6.1	58.7±5.5	rt-PA	rt-PA 0.9 mg/kg, iv,qd	rt-PA	rt-PA 0.6 mg/kg, iv,qd	2 weeks	①③
Wang FP	2024	36	(20:16)	36	(25:11)	67.44±6.14	67.06±6.22	rt-PA	rt-PA 0.9 mg/kg, iv,qd	rt-PA	rt-PA 0.6 mg/kg, iv,qd	2 weeks	①
Yu M	2009	15	(8:7)	15	(10:5)	68±10	68±11	rt-PA	rt-PA 0.9 mg/kg, iv,qd	rt-PA	rt-PA 0.6 mg/kg, iv,qd	2 weeks	①
Yu M	2019	38	(23:15)	40	(25:15)	84.95±2.49	85.76±2.34	rt-PA	rt-PA 0.9 mg/kg, iv,qd	rt-PA	rt-PA 0.6 mg/kg, iv,qd	2 weeks	①
Zhang Z	2022	48	(26:22)	48	(27:21)	59.37±5.62	60.05±5.78	rt-PA	rt-PA 0.9 mg/kg, iv,qd	rt-PA	rt-PA 0.6 mg/kg, iv,qd	2 weeks	①

*①NIHSS ; ②BI; ③adverse effects rate; po: oral administration; qd: once a day; bid: twice a day; tid: three times a day; NBP: Dl-3n-butylphthalide; HUK: Human urinary kallidinogenase; EDB: Edaravone Dexborneol; IG: intervention group; CG: control group; rt-PA: recombinant tissue plasminogen activator; NA Not applicable
